# Probing the distinct chemosensitivity of *Plasmodium vivax* liver stage parasites and demonstration of 8-aminoquinoline radical cure activity in vitro

**DOI:** 10.1038/s41598-021-99152-9

**Published:** 2021-10-07

**Authors:** Steven P. Maher, Amélie Vantaux, Victor Chaumeau, Adeline C. Y. Chua, Caitlin A. Cooper, Chiara Andolina, Julie Péneau, Mélanie Rouillier, Zaira Rizopoulos, Sivchheng Phal, Eakpor Piv, Chantrea Vong, Sreyvouch Phen, Chansophea Chhin, Baura Tat, Sivkeng Ouk, Bros Doeurk, Saorin Kim, Sangrawee Suriyakan, Praphan Kittiphanakun, Nana Akua Awuku, Amy J. Conway, Rays H. Y. Jiang, Bruce Russell, Pablo Bifani, Brice Campo, François Nosten, Benoît Witkowski, Dennis E. Kyle

**Affiliations:** 1 Center for Tropical and Emerging Global Diseases, University of Georgia, 500 DW Brooks Dr. Suite 370, Athens, GA 30602 USA; 2 Malaria Molecular Epidemiology Unit, Institut Pasteur du Cambodge, 5 Boulevard Monivong, PO Box 983, Phnom Penh, 12201 Cambodia; 3 Shoklo Malaria Research Unit, Mahidol-Oxford Tropical Medicine Research Unit, Faculty of Tropical Medicine, Mahidol University, 68/30 Bantung Rd., Mae Sot, Tak, 63110 Thailand; 4 Centre for Tropical Medicine and Global Health, Nuffield Department of Medicine Research Building, University of Oxford, Old Road Campus, Oxford, UK; 5 Infectious Diseases Laboratories (ID Labs), Agency for Science, Technology and Research (A*STAR), Immunos, Biopolis, Singapore, 138648 Singapore; 6 Department of Microbiology and Immunology, University of Otago, Dunedin, New Zealand; 7 Medicines for Malaria Venture (MMV), Route de Pré-Bois 20, 1215 Geneva, Switzerland; 8 Department of Global Health, College of Public Health, Center for Global Health and Infectious Disease Research, University of South Florida, 3720 Spectrum Blvd Suite 402, Tampa, FL 33612 USA; 9 Department of Microbiology and Immunology, Yong Loo Lin School of Medicine, National University of Singapore, Singapore, 117545 Singapore; 10 Faculty of Infectious and Tropical Diseases, London School of Hygiene and Tropical Medicine, London, WC1E 7HT UK

**Keywords:** Biological techniques, Drug discovery, Diseases

## Abstract

Improved control of *Plasmodium vivax* malaria can be achieved with the discovery of new antimalarials with radical cure efficacy, including prevention of relapse caused by hypnozoites residing in the liver of patients. We screened several compound libraries against *P. vivax* liver stages, including 1565 compounds against mature hypnozoites, resulting in one drug-like and several probe-like hits useful for investigating hypnozoite biology. Primaquine and tafenoquine, administered in combination with chloroquine, are currently the only FDA-approved antimalarials for radical cure, yet their activity against mature *P. vivax* hypnozoites has not yet been demonstrated in vitro*.* By developing an extended assay, we show both drugs are individually hypnozonticidal and made more potent when partnered with chloroquine, similar to clinically relevant combinations. Post-hoc analyses of screening data revealed excellent performance of ionophore controls and the high quality of single point assays, demonstrating a platform able to support screening of greater compound numbers. A comparison of *P. vivax* liver stage activity data with that of the *P. cynomolgi* blood, *P. falciparum* blood, and *P. berghei* liver stages reveals overlap in schizonticidal but not hypnozonticidal activity, indicating that the delivery of new radical curative agents killing *P. vivax* hypnozoites requires an independent and focused drug development test cascade.

## Introduction

Malaria is a significant global health problem^1^. Human malaria is caused by five different species of the *Plasmodium* parasite, including *Plasmodium vivax*, the most widespread, and *P. falciparum*, the most virulent^2^. As control efforts are reducing falciparum malaria incidence worldwide, controlling vivax malaria is lagging behind^3^. Upon infection of a new host, *Plasmodium* parasites must complete an asymptomatic and undetectable liver infection prior to initiating a symptomatic and transmittable blood infection. Contributing to the lag in vivax control are hypnozoites remaining in the liver of infected patients; these treatment-insensitive forms resume development days, weeks, or even months after the primary infection, causing a secondary ‘relapse’ infection and transmission^4^. Today, vivax malaria is an economic burden in developing countries, as well as an encumbrance to the livelihood of the people it afflicts^5,6^.

There are currently only two FDA-approved drugs for the radical cure of vivax malaria, including relapse prevention, namely, the 8-aminoquinolines (8AQs) primaquine and tafenoquine, which must be co-administered with chloroquine. Chloroquine is a blood schizonticide that has been shown to synergize with 8AQs for radical cure efficacy^7,8^, but is now compromised by geographical resistance^9–11^. The potential of 8AQs is further mired by contraindications, such as for glucose-6-phosphate deficient (G6PD) individuals^12^. Of note, tafenoquine must be administered with a quantitative test for G6PD and is not currently approved for use with children or pregnant women due to hemolytic potential^13^. As such, progressing new drugs for treatment of vivax malaria and prevention of relapse are important areas of antimalarial drug discovery and development^14^.

Studying *Plasmodium* liver stages requires intricate logistics, expensive materials, and advanced techniques; several recent reports describe critical advancements in this field which have enabled deeper investigation of the liver stages of *P. vivax* and *P. cynomolgi* (a relapsing species closely related to *P. vivax*)^15–20^*.* Despite these advances, the challenge of studying hypnozoites has left critical questions unanswered. First, for decades, discovery of new antimalarial drugs has been performed with well-established cultures models such as the blood stage of *P. falciparum*^21^ and the liver stage of rodent malaria species such as *P. berghei*^22^. Such models have enabled high-throughput screening leading to novel antimalarial development, such as the imidazolopiperazine KAF156^23,24^. However, these models focus on inhibition of the rapid growth of asexually replicating blood and liver schizonts. As a result, antimalarials currently under development do not address hypnozoite biology, which is defined by long-term quiescence involving mechanisms and phenotypes starkly different than that of a rapidly growing schizont^25^. Thus, there are many compounds with known activity against other *Plasmodium* stages and species for which an understanding of hypnozonticidal activity would greatly help prioritize development. Second, it has been suggested that a pre-screen using one or more of the aforementioned culture models could be utilized as a rapid and inexpensive method to generate a compound library enriched for hypnozonticidal activity, but this hypothesis has not been tested^22,26^.

**Figure 1:**
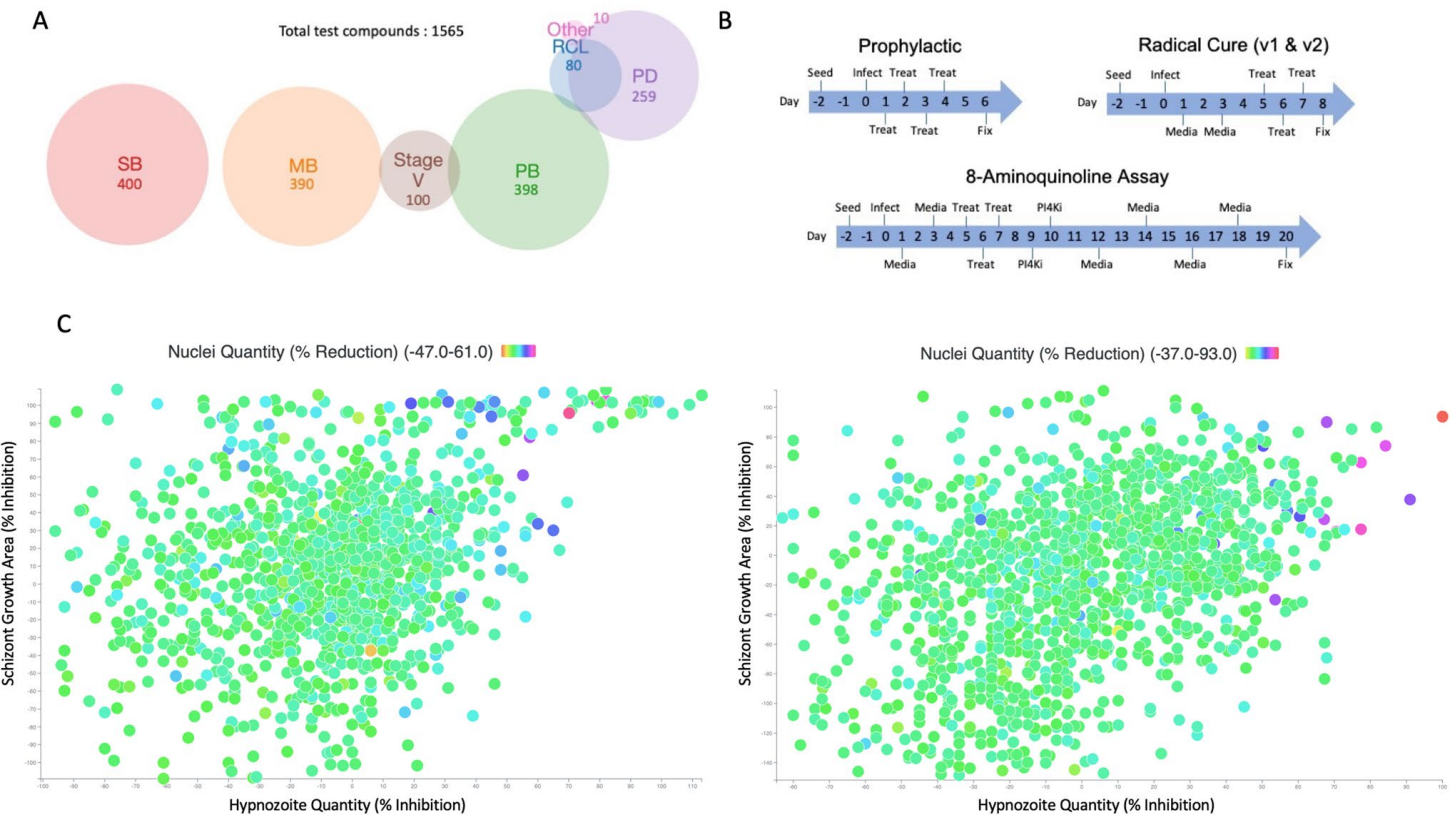
Library contents, assay workflow, and Single Point data summary. (**A**) Venn diagram of all compounds tested, grouped by library. RCL, Reference Compound Library; PB, Pathogen Box; SB, Stasis Box; MB, Malaria Box; PD, Photodiversity Library. (**B**) Summary of assay workflow for Prophylactic and Radical Cure modes as well as a newly-developed 20-day assay to assess activity for 8-aminoquinolines. Media indicates media change, Treat indicates a media change followed by treatment with compounds via pin tool, PI4Ki indicates addition of 1 μM MMV390048 added to the culture during media change. (**C**) All SP data from the highest-quality Prophylactic runs (left) and 8-day Radical Cure runs (right). X-axis represents inhibition of hypnozoite quantity, Y-axis represents inhibition of schizont growth, color represents reduction of hepatic nuclei, likely due to toxicity. For nuclei reduction, green and blue represent little or no loss of nuclei (< 10%), deep blue represents some loss of nuclei (10–30%), purple and red represent dramatic loss of nuclei (> 30%). Control well data were removed for better visualization.

To begin addressing these questions, we screened several small compound collections against *P. vivax* liver stages (PvLS), including the MMV Reference Compound Library, Pathogen Box^27^, Stasis Box, Malaria Box^28^, Photodiversity Library, and a collection of 100 compounds with activity against *P. falciparum* stage V gametocytes^27,29–32^, with a focus on discovering compounds with activity against mature hypnozoites (Fig. 1A). While our screening platform did identify new hypnozonticidal compounds, our standard 8-day assay format did not detect 8AQ activity against mature hypnozoites, similar to reports from us and others^18,19,33^. We therefore developed an assay format with an extended endpoint which not only allows us to observe activity of 8AQs against mature hypnozoites in vitro, but also allows us to demonstrate synergy with the partner drug chloroquine; a useful tool for studying the mechanisms of these drugs. To assess the predictive value of alternative screening platforms, we collected activity data from previously-published screens and supplemented this dataset by screening the *P. cynomolgi* asexual blood stage against the Reference Compound Library^34^. Post-hoc analyses of discovery rates reveals mature hypnozoites are broadly insensitive to compounds with schizonticidal activity. As other screening platforms do not predict hypnozonticidal activity, herein we report assay refinements and a screen cascade to increase the predictive value and efficiency of our PvLS platform.

## Results

### General overview of assay modes, libraries, and assay refinement

Currently defined as a uninucleate form of similar size to the host hepatocyte nucleus, *P. vivax* hypnozoites are known to be sensitive to treatment with several newly developed antimalarials during the first few days after hepatocyte invasion, but are insensitive to these compounds once reaching full maturation between days 4–6 post-infection^35–37^. Critically, characterizing the radical cure activity of compounds is dependent on testing mature hypnozoites, and the first in vitro assays to demonstrate medium throughput as such were only recently reported^18,19,38^. These reports describe two distinct in vitro assay modes: Prophylactic mode, in which *P. vivax* liver stages are treated before or shortly after hepatocyte infection to assess the sensitivity of early schizonts and immature hypnozoites, and Radical Cure mode, in which liver stages are treated at 5 days post-infection in order to evaluate the sensitivity of late schizonts and mature hypnozoites (Fig. 1B). Prophylactic and Radical Cure single point (SP) runs were performed for the Reference Compound Library (RCL), Pathogen Box (PB), Stasis Box (SB), and Malaria Box (MB), but to manage limited throughput, hit confirmation by dose response (DR) was primarily performed for Radical Cure hits. Likewise, only Radical Cure runs were performed for the Photodiversity Library (PD), a diverse collection of 259 compounds assembled by the University of Sussex and MMV. Additionally, several antibiotics (supplementing those in the RCL) were run in Prophylactic and Radical Cure DR assays, while several ionophores and a collection of 100 compounds found active against *P. falciparum* stage V gametocytes were assessed in Radical Cure DR assays. Taking into consideration library overlap and batch-specific testing, our SP Radical Cure screen included 1527 test wells containing 1478 unique batches of 1465 unique structures while our SP Prophylactic screen included 1270 test wells containing 1270 unique batches of 1265 unique structures. In all, we tested 1565 compounds in some capacity. A summary of library contents, testing modes, and SP screen data is shown in Fig. 1.

**Figure 2:**
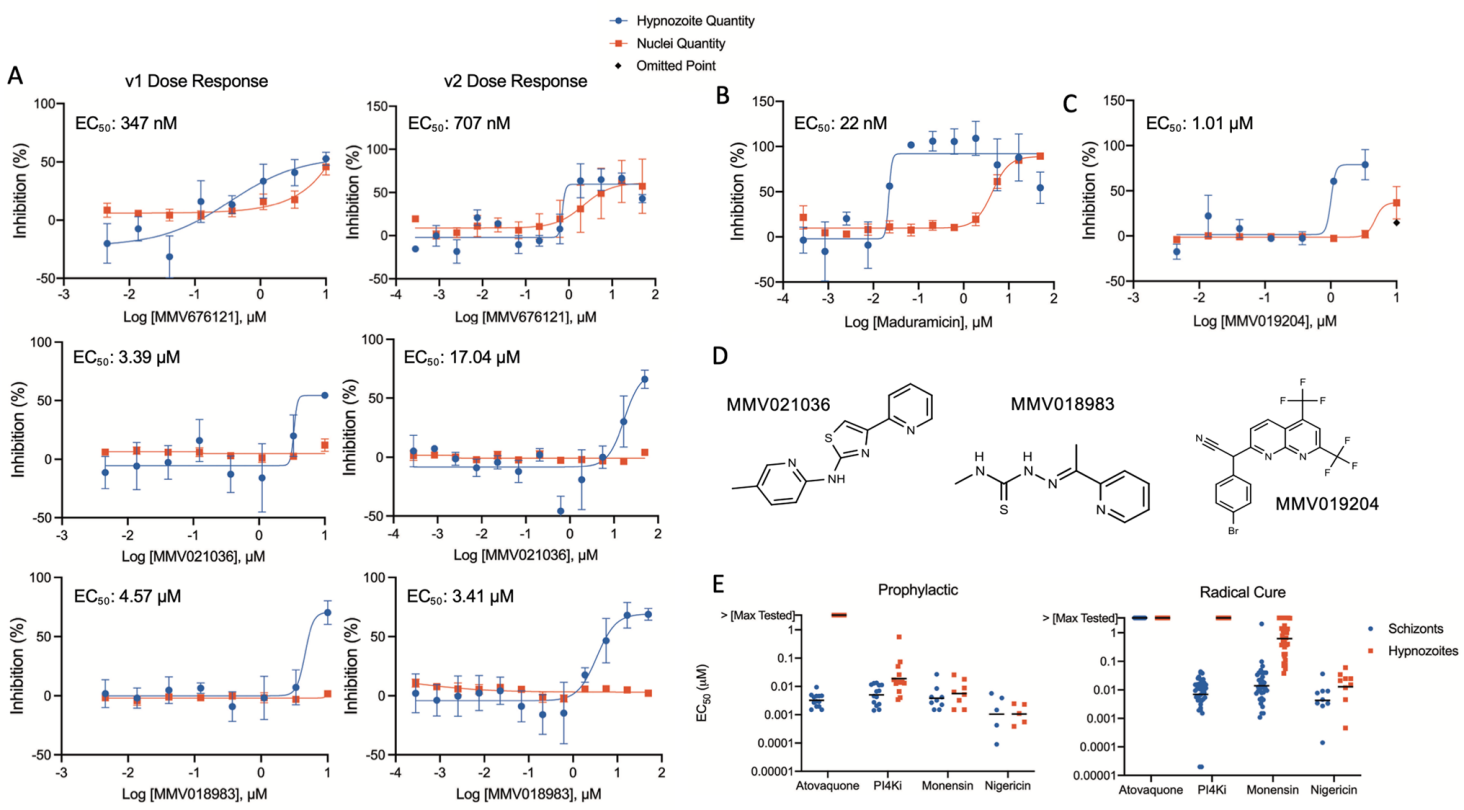
Examples of 8-day Radical Cure assay improvements used to characterize hits with activity against mature hypnozoites. (**A**) EC_50_’s shown are for inhibition of hypnozoites. Possible hit MMV676121 was first identified from a run of the original dose response assay, which included a concentration gradient from 10 μM to 46 nM (v1). A CC_20_ could not be calculated for inhibition of hepatic nuclei but was estimated to be > 2 μM. Bars represent SEM of 6 independent experiments, each with a single well at each concentration. MMV676121 was retested in the refined (v2) assay dose response format, which includes an expanded concentration gradient from 50 μM to 280 pM. Bars represent SEM of two independent experiments, each containing two wells at each concentration. Plotting inhibition of hepatic nuclei on the hypnozoite inhibition chart shows no selectivity window for this compound. The two assay versions were also used to identify and then further define the selective activity of two *P. falciparum* stage V gametocyte inhibitors, MMV021036 and MMV018983, against mature hypnozoites. Bars in the v1 charts represent SEM of three (for MMV021036) or four (for MMV018983) independent experiments, each with a single well at each concentration; bars in the v2 charts represent SEM of two independent experiments, each containing two wells at each concentration. (**B**) Radical Cure activity from a v2 assay of maduramicin against hypnozoites. Maduramicin is potently active against hypnozoites (EC_50_ 22 nM) and toxic against host hepatocytes (EC_50_ 4.28 μM), leading to a calculated selectivity of > 190. Bars represent SEM of two independent experiments, each containing two wells at each concentration. (**C**) Radical Cure activity from a v2 assay of *P. falciparum* stage V gametocyte inhibitor MMV019204, with activity against hypnozoites (EC_50_ 1.01 μM) and toxicity against host hepatocytes (EC_50_ 4.71 μM), leading to a calculated selectivity of 4.7. Black marker indicates hypnozoite inhibition data marked as an outlier. Bars represent SEM of two independent experiments, each containing two wells at each concentration. (**D**) Structures of the three *P. falciparum* stage V gametocyte inhibitors with activity against mature hypnozoites. (**E**) Plots of all EC_50_’s for the control and reference compounds of Prophylactic and Radical Cure dose response assays used to generate data for this report. Dose response assays were performed at two sites (SMRU in Thailand and IPC in Cambodia) over 3 years using three different lots of human hepatocytes (UBV, PDC, and BGW) infected with sporozoites from a unique patient isolate of *P. vivax* for each run*.* For visualization, data was plotted at 3.3 μM for all runs in which activity was greater than the maximum concentration tested (10 μM for atovaquone or 3.3 μM for PI4Ki and monensin). Bar represents geometric mean.

After analyzing the performance of our previously-described (v1) DR assay^19^, we decided to increase DR assay sensitivity at the cost of throughput by expanding the dose range from 8 points (10 μM–46 nM) to 12 points (50 μM–280 pM), as well as change from singleton to duplicate wells at each concentration (Fig. S1B). This refined assay format (v2) resulted in better resolution of PvLS activity and hepatic toxicity, as noted with compound MMV676121 (Fig. 2A). This compound was originally detected as a hit in a v1 DR assay and, following several confirmation runs, was thought to be the first new hypnozonticial hit discovered from our screen. As follow up, a set of 15 analogs of MMV676121 were assayed to provide an initial structure–activity relationship dataset, but none of the analogs were found selectively active. This lack of activity was explained when we retested MMV676121 in the v2 assay in which it was clarified as nonspecific. We noted loss of hepatic nuclei at all doses that also showed activity against hypnozoites (from 1.85 μM), thus even though comparing EC_50_’s for hypnozonticidal activity (707 nM) and toxicity (2.17 μM) results in a calculated selectivity index of ~ 3, these results show concentrations resulting in 15–20% reduction in hepatic nuclei are not indicating parasite-specific activity. We therefore began quantifying toxicity in all DR runs by establishing a 20% reduction in hepatic nuclei as a threshold for a calculation of toxicity (CC_20_), often using operators (i.e. > highest unaffected concentration) as many toxicity curves were either partial or non-sigmoidal. Similarly, the 100 *P. falciparum* stage V gametocyte inhibitors were first tested in the v1 Radical Cure assay, and then hits were confirmed in the v2 assay, which helped resolve the potency and selectivity of MMV018983 (EC_50_ 3.41 μM) and MMV021036 (EC_50_ 17.04 μM) against mature hypnozoites (Fig. 2A, D). Furthermore, we noted that very often at toxic concentrations the ability of attenuated PvLS parasites to clear from wells was diminished, possibly due to the loss of hepatocyte-driven clearance mechanisms such as autophagy^39^. These PvLS parasites were quantified during image analysis and resulted in a bell-shaped dose response curve, which often required masking of the parasite inhibition values of toxicity-affected doses in order to enable a proper curve fit. The ionophore maduramicin is an example; in our refined v2 assay we noted a full sigmoidal dose response curve against mature hypnozoites (EC_50_ 22 nM) including toxicity and loss of hypnozoite clearance at higher doses (EC_50_ 4.28 μM), resulting in a selectivity window of > 190 (Fig. 2B). These methods to remove toxic outliers and resolve selectivity were used to determine the narrow but possibly selective activity of the *P. falciparum* stage V gametocyte inhibitor MMV019204 against mature hypnozoites (Fig. 2C, D).

Because the 8AQs are, in combination with chloroquine, the only FDA-approved drugs for radical cure treatment of *P. vivax*, they are perhaps the most suitable positive control for in vitro radical cure drug discovery. However, as shown in our previous report^19^, primaquine and tafenoquine were found inactive or weakly active in 8-day Radical Cure assays and therefore could not be used as positive controls. While a screen can be performed without a positive control, in which case activity data is normalized to negative controls or Z factor^40^, we found the ionophores monensin^19^ and nigericin were potent, selective, and able to rapidly clear mature hypnozoites by day 8 post-infection. Therefore, ionophore positive controls were incorporated into our platform as assay development progressed (Fig. 2E, S3A). Interestingly, when we exhausted our supply of cryopreserved human hepatocytes and switched lots, we noted diminished activity of monensin against mature hypnozoites, likely due to greater metabolic activity in the new lot as noted from quality-control data from the vendor. We then began using nigericin as the positive control for all DR assays and found it reproducibly and potently active (geometric mean EC_50_ against hypnozoites in Radical Cure assays: 13 nM, n = 9, Fig. 2E).

### Screening the reference compound library in prophylactic and radical cure modes

The RCL is a collection of 80 newly deployed, developmental, or legacy antimalarials used to validate and characterize MMV-supported small molecule screening platforms. We performed Prophylactic and Radical Cure mode SP assays with the RCL, followed by DR confirmation of hits. As noted in Tables 1 and 2, the four compounds we found most potent against immature hypnozoites and schizonts were phosphatidylinositol 4-kinase (PI4K) inhibitors UCT943 and CC_642944^41^, eukaryotic elongation factor 2 inhibitor DDD107498^42^, and KAF156^24^. All four compounds exhibited low nanomolar potency, in agreement with the relative potency range previously described in a Prophylactic assay^19^*.* While an additional 29 RCL compounds were active against either early or late schizonts, none of the 80 tested, including the 8AQs, were found active against mature hypnozoites in our 8-day Radical Cure assay mode (Tables 1, 2; Fig. S2A, B).

**Table 1:** Confirmed hits from 6-day prophylactic assays of the reference compound library.

Synonyms	Class or target^REF^	Schizont net growth area		Hypnozoite quantity		Nuclei quantity	
		EC_50_ (μM)	Max Inh (%)	EC_50_ (μM)	Max Inh (%)	CC_20_ (μM)	Max Inh (%)
UCT943	PI4K^41^	0.00175	100	0.00427	100	> 10.0	15.6
CC_642944	PI4K^41^	0.00476	101	101	98.9	> 10.0	1.28
DDD107498 (M5717)	eEF2^42^	0.00257	101	0.0104	100	> 10.0	0.326
KAF156	IZP^24^	0.0141	103	0.0329	105	> 10.0	4.83
Clindamycin	Antibiotic^43^	0.00597	79.5	> 50.0	53.8	> 50.0	5.01
Methotrexate	Folic acid antagonist^44^	0.0428	99.1	> 10.0	12.9	> 10.0	6.07
P218	DHFR^45^	0.0511	101	> 10.0	5.15	> 10.0	9.08
ELQ-300	Cytochrome bc1^46^	0.0576	97.9	> 1.11	71.9	> 10.0	7.2
DSM265	DHODH^47^	0.126	100	> 3.07	37.4	> 3.07	9.06
DSM421	DHODH^48^	0.325	98.8	> 10.0	61.2	> 10.0	4.76
AZ412	V-type H+-ATPase^49^	0.686	99.7	> 3.33	54.8	> 10.0	2.49
Cycloheximide	Antifungal^50^	1.39	99.6	> 3.33	85.7	> 10.0	8.17
AN13762 (AN762)	Oxaborole, CPSF3^51^	1.63	99.7	> 1.11	93.2	> 10.0	6.64
Chlorproguanil	DHFR^52^	> 1.11	98.1	> 10.0	26	> 10.0	9.62
NPC-1161B	8AQ^53^	> 0.370	91.9	> 10.0	41.3	> 10.0	6.73
Pamaquine	8AQ^54^	ND	87.1	> 3.33	27.1	> 10.0	1.25
*N*-Desethylamodiaquine	4AQ^55^	> 3.33	74.5	> 10.0	15.8	> 10.0	15.3
*Trans*-Mirincamycin	Antibiotic^56^	ND	72.5	> 10.0	47.4	> 10.0	4.62
*Cis*-Mirincamycin	Antibiotic^56^	ND	69.3	> 10.0	19.5	> 10.0	13.2
Doxycycline hydrochloride	Antibiotic^57^	> 0.370	68.3	> 10.0	36.7	> 10.0	3.57
Amodiaquine	4AQ^55^	0.0141	65.6	> 10.0	16.3	> 10.0	18.6
Azithromycin	Antibiotic^58^	> 0.370	63.3	> 10.0	24.6	> 10.0	10.7
Artemether	Artemisinin derivative^59^	ND	60.4	> 10.0	35.2	> 10.0	11.4
Tetracycline	Antibiotic^57^	ND	56.7	> 10.0	19.9	> 10.0	3.64
AQ-13	4AQ^60^	ND	56.1	> 10.0	29.4	> 10.0	6.97

All EC_50_’s, CC_20_’s, and Maximum Inhibition (Max Inh) values are from the highest quality independent experiment. RCL, Reference Compound Library; PI4K, phosphatidyl inositol 4-kinase; eEF2, elongation factor 2; IZP, imidazolopiperazine; DHFR, dihydrofolate reductase; DHODH, dihydroorotate dehydrogenase; CPSF3, cleavage and polyadenylation specificity factor subunit 3; 8AQ, 8-aminoquinoline; 4AQ, 4-aminoquinoline. Included are activity values for selective compounds; activity data for selective and nonselective compounds, as well as structures, can be found in Supplementary Table S1 online.

**Table 2:** Confirmed hits from 8-day radical cure assays.

Synonyms	Library or collection: class, target, or disease set^REF^	Schizont net growth area		Hypnozoite quantity		Nuclei quantity	
		EC_50_ (μM)	Max Inh (%)	EC_50_ (μM)	Max Inh (%)	CC_20_ (μM)	Max Inh (%)
Maduramicin	Ionophore: Coccidiostat^61^	0.000705	101	0.0225	120	2.59	89.6
Narasin	Ionophore: Coccidiostat^62^	0.0075	101	0.0646	125	8.8	58.9
Salinomycin	Ionophore: Coccidiostat^62^	0.0402	102	0.112	104	> 10.0	9.18
Lasalocid-A	Ionophore: Coccidiostat^62^	0.0431	100	1.05	78.9	> 10.0	9.93
MMV019204	Stage V	1.24	86	0.957	62.2	5.44	54.7
MMV018983	Stage V	4.44	100	1.92	82.8	> 50.0	10.9
MMV021036	Stage V	ND	90.9	19.9	71	> 50.0	7.93
CC_642944	RCL: Antimalarial, PI4K^41^	0.0239	96.4	> 10.0	6.87	> 10.0	12.1
Cycloheximide	RCL: Antifungal^50^	0.0856	96.3	> 10.0	− 3.77	> 10.0	3.7
KAF156	RCL: Antimalarial, IZP^24^	0.33	93.2	> 1.00	52.2	> 1.00	3.09
AZ412	RCL: Antimalarial, V-type H+-ATPase^49^	0.381	104	> 10.0	29.4	> 10.0	9.37
Thiostrepton	RCL: Antibiotic, rRNA inhibitor^63^	> 3.33	80.2	> 10.0	11.3	> 10.0	4.33
Ro 47-7737	RCL: Antimalarial, 4AQ^64^	> 3.33	70.6	> 10.0	2.26	> 10.0	10.1
DSM265	RCL: Antimalarial, DHODH^47^	> 1.11	69.4	> 10.0	0.114	> 10.0	9.12
SJ733	RCL: Antimalarial, ATP4^65^	ND	56.8	> 50.0	21	> 50.0	8.19
Arterolane, OZ277	RCL: Antimalarial, Artemesinin analog^66^	> 10.0	51.4	> 10.0	40.7	> 10.0	6.82
MMV085499	PB: Malaria, PI4K^67^	0.0012	85.1	> 50.0	24.7	> 50.0	10.3
DDD00108451, MMV667494	PB: Malaria, eEF2^68^	0.00316	103	> 50.0	21.7	> 50.0	14.5
MMV026356	PB: Malaria^69^	0.0266	103	> 50.0	40.9	> 50.0	− 0.938
MMV668727	PB: Onchocerciasis	0.19	87.5	> 50.0	50.1	> 50.0	10.2
MMV675993	PB: Cryptosporidiosis	0.294	71.3	> 50.0	14.5	> 50.0	7.81
MMV026020	PB: Malaria	0.418	99.2	> 50.0	31.4	> 50.0	7.48
MMV023860	PB: Malaria	0.57	88.2	> 50.0	− 3.82	> 50.0	10.1
MMV024443	PB: Malaria, CDPK1^24^	3.11	84.7	> 50.0	38.5	> 50.0	28.2
MMV688980	PB: Malaria, ATP4^70^	> 16.7	87.6	> 50.0	40.3	> 50.0	1.44
MMV010576	PB: Malaria, Kinase inhibitor^67^	> 16.7	80.2	> 50.0	17.9	> 50.0	23.6
MMV010545	PB: Malaria, CDPK1 or PK7^67^	ND	79.2	> 50.0	40.3	> 50.0	38.1
MMV688548	PB: Toxoplasmosis	> 16.7	76.7	> 50.0	20.4	> 50.0	9.03
MMV023370, MMV676359	PB: Malaria	> 50.0	63.7	> 50.0	36.8	> 50.0	14
MMV690491	SB	0.395	99.2	> 10.0	32.4	> 10.0	3.96
MMV690648	SB	1.23	99	> 10.0	41.5	> 10.0	10.2
MMV396797	MB: Drug like, Heme catabolism^71^	0.599	95.7	> 50.0	− 6.1	> 50.0	12.7
MMV019266	MB: Drug like, Heme catabolism^71^	1.62	92.9	> 50.0	31.3	46.9	48.8
MMV1091186	Stage V^22^	2.86	91	> 50.0	24.8	> 50.0	27.5
MMV019861	Stage V	> 5.56	86.8	> 50.0	38.9	> 16.7	12.7
MMV516035	Stage V	ND	80.2	> 50.0	48.3	> 50.0	7.78
MMV008470	Stage V	> 3.33	72.6	> 10.0	62	> 10.0	3.51
MMV1022644	Stage V	> 3.33	64.7	> 10.0	7.41	> 10.0	0.357
MMV335848	Stage V	ND	60.5	> 10.0	− 9.9	> 10.0	13.5
Ciprofloxacin	Antibiotic^72^	> 10.0	59.2	> 10.0	36.3	> 10.0	21.2
Ceftazidime pentahydrate	Antibiotic^73^	> 10.0	47.9	> 10.0	38.3	> 10.0	22

All EC_50_’s, CC_20_’s, and Maximum Inhibition (Max Inh) values are from the highest quality independent experiment. RCL, Reference Compound Library; PB, Pathogen Box; SB, Stasis Box; MB, Malaria Box; Stage V, *P. falciparum* stage V gametocyte active; eEF2, elongation factor 2; PI4K, phosphatidyl inositol 4-kinase; IZP, imidazolopiperazine; DHFR, dihydrofolate reductase; DHODH, dihydroorotate dehydrogenase; ATP4, ATPase 4; CDPK1, calcium-dependent protein kinase 1; PK7, protein kinase domain-containing protein 7; PKG, cyclic GMP-dependent protein kinase. Included are activity values for selective compounds; activity data for selective and nonselective compounds, as well as structures, can be found in Supplementary Table S2 online.

### Radical cure confirmed activity summary

To manage available throughput, the majority of SP screening and DR confirmation were performed using the Radical Cure assay mode (Table 2). Building off our previous report on the Radical Cure hypnozonticial activity of monensin as well as Prophylactic activity of other ionophores including nigericin, salinomycin, and lasalocid-A^19^, we tested these and several other ionophores in Radical Cure assays and found potent hypnozonticidal activity across this class of compounds. Four ionophores were found much more potent against mature hypnozoites than monensin: maduramicin (EC_50_ 710 pM), narasin (EC_50_ 7.5 nM), nigericin (EC_50_ 13 nM), and salinomycin (EC_50_ 40 nM). Other than ionophores, of the 1565 compounds tested in Radical Cure assays in this report, the only novel hypnozonticidal activity found was from three of 100 compounds found active against *P. falciparum* stage V gametocytes: MMV018983, MMV021036, and MMV109204 (Fig. 2A, D). However, our Radical Cure cascade did for the first time characterize the sub-micromolar potency of several compounds or scaffolds previously unknown to have activity against late *P. vivax* liver schizonts, including cycloheximide, AZ412, MMV026356, MMV668727, MMV675993, MMV026020, and MMV023860 (Tables 2, S2).

### Demonstration of 8-aminoquinoline activity and synergy with chloroquine

**Figure 3:**
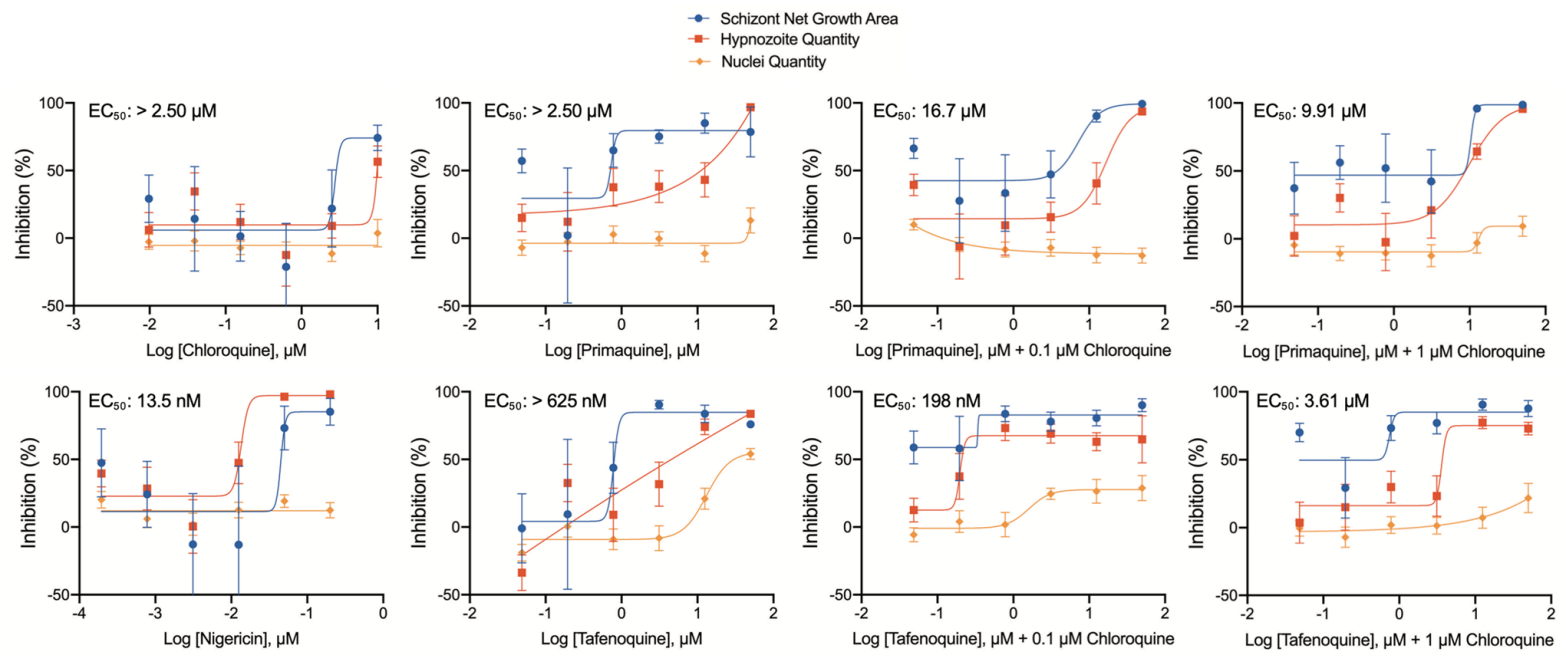
Demonstration of 8AQs synergy with chloroquine in vitro using a 20-day assay. Chloroquine, primaquine and tafenoquine were assayed in the 20-day assay format alone and in combination with chloroquine as indicated on the X-axis. Chloroquine was found slightly toxic at 10 μM, thus the 0.1 and 1 μM combinations with 8AQs are shown. Nigericin was ran in all assays as a positive and normalization control. EC_50_ shown indicates potency against hypnozoites. Bars represent the SEM of three independent experiments; the first two experiments were the average of two replicate wells for each concentration, the third experiment was the average of four replicate wells for each concentration.

As demonstrating 8AQ activity in vitro is an important prerequisite for studying the peculiar and elusive mechanisms of these drugs^74^, and they tested negative in our 8-day Radical Cure assay (Fig. S2A, B), we developed a unique assay mode specifically for 8AQs. Several reports describe the synergistic effect of chloroquine on 8AQ activity against *P. vivax *in vivo^75^, as well as *P. cynomolgi *in vivo^8^, and recently in vitro^76^, thus we hypothesized synergy could be an important element to incorporate into our PvLS platform. Furthermore, we and others have reported 8AQ-treated PvLS take excessive time to clear from culture^18,33,77^, thus we re-designed the Radical Cure assay to run for 12 and 20 days post-infection and included chloroquine, primaquine, and tafenoquine alone, in addition to primaquine and tafenoquine in co-treatment with 0.1, 1, or 10 μM chloroquine. Cultures were treated with 1 μM PI4K inhibitor MMV390048 on days 9 and 10 post-infection, 2 days after finishing a 3-day treatment with test compound, to clear schizonts from the culture^18^ (Fig. 1B). In both the 12- and 20-day assays, primaquine and tafenoquine produced active, yet non-sigmoidal, DR curves against mature hypnozoites (Fig. 3, S2B). However, the 20-day assay produced DR curves with the highest maximum inhibition and most potency, especially with the addition of chloroquine (Fig. 3). In particular, primaquine with 0.1 and 1 μM chloroquine resulted in an EC_50_ against mature hypnozoites of 16.7 μM and 9.91 μM, respectively, while 0.1 μM chloroquine with tafenoquine, with an EC_50_ of 198 nM, was found the most potent hypnozonticidal combination.

### Run quality, cutoff, predictive value, and toxicity

We performed several post-hoc analyses assessing the predictive value of the assay to understand how to better structure the screening cascade to minimize the false discovery rate (FDR). We have previously reported run quality is largely driven by infection rate (PvLS per well), which currently cannot be predicted from run-to-run but can be generally increased by infecting wells with an ample quantity of sporozoites (1–2 × 10^-4^) and validated hepatocyte lots^19^. We found an infection rate of 40–50 hypnozoites per well was sufficient to generate a Z′ factor of 0.0–0.5 (Fig. S3B), or enough confidence to simply designate compounds as ‘yes/no’ with a manageably low FDR which can then be resolved during DR confirmation^78^. To demonstrate how FDR changes in high- versus low-quality assays (as determined by Z′ factor), inhibition data from two SP runs of the same library were collected and charted together (Fig. S3C). In all multi-run comparisons, the positive control consistently exhibited a narrow deviation around 100% inhibition, while the deviation around the DMSO negative control mean narrowed in the superior run as compared to the inferior run. Thus, despite the variability noted in inactive or untreated wells, we determined our assay consistently detected completely active compounds, such as those with an activity profile like ionophores. However, because we detected a range of partial-actives (with inhibition between 60 and 75%) and large inactivity window (Figs. S4, S5, S6) we found even a SP run with a robust Z′ factor alone could not provide a precise inhibition cutoff for selecting putative hits for confirmation.

**Figure 4:**
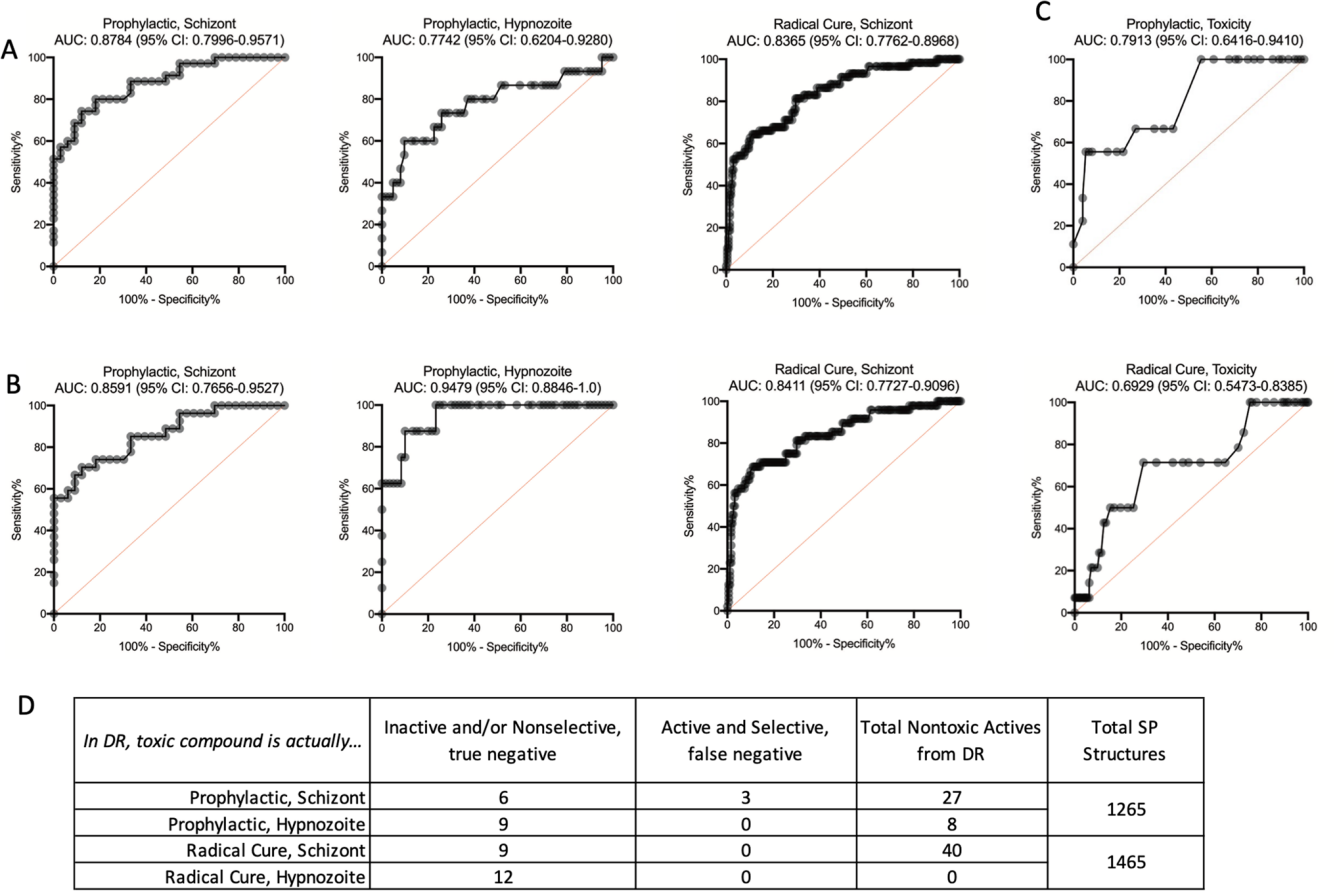
Receiver-operator characteristic (ROC) analyses of screen data. ROC curves were calculated for all compounds from the Reference Compound Library, Malaria Box, Pathogen Box, and Stasis Box for which dose response (DR) data were collected as part of this report, area under curve (AUC) and 95% CI are indicated. (**A**) ROC curves of Prophylactic or Radical Cure activity against schizonts or hypnozoites for compounds exhibiting unambiguously active or inactive curves in DR assays, regardless of possible toxicity. (**B**) ROC curves of Prophylactic or Radical Cure activity against schizonts or hypnozoites for compounds exhibiting unambiguously active or inactive curves and no toxicity at 10 μM in DR assays. ROC curves were not calculated for Radical Cure activity against hypnozoites because no hits were found from SP assays. (**C**) ROC curves of compounds found toxic at 10 μM in Prophylactic and Radical Cure DR assays. (**A**–**C**) Red line indicates a random assay. (**D**) Table quantifying all compounds considered putative hits in SP assays which were found toxic at 10 μM in DR confirmation assays. Only 3 compounds with Prophylactic activity against schizonts exhibited a selectivity window in DR confirmation, all other compounds did not exhibit selective activity.

To better understand how discovery rates were affected by different cutoffs, the SP and DR data were subjected to Receiver-Operator Characteristic (ROC) analyses^79^. As noted above, toxic compounds often resulted in ambiguous activity at toxic doses. These compounds contribute to the FDR as they can appear active in the SP assay but show non-selectivity when tested in DR (i.e. pyronaridine, Table S1), or they can contribute to the False Omission Rate (FOR) when PvLS are not cleared at toxic doses, (i.e. MMV019204, Fig. 2C). As our screen cascade lacked a method for a priori detection of toxicity, the first ROC analysis included all screen data to assess sensitivity and specificity when toxic or nonselective compounds were included (Fig. 4A), while the second ROC analysis only included compounds found nontoxic at 10 μM in DR assays, to understand the impact of toxic and nonspecific compounds on discovery rates (Fig. 4B). Removal of toxics resulted in better predictive value for most endpoints (for example, AUC increased from 0.7742 to 0.9479 for hypnozoite activity in Prophylactic assays, Fig. 4B), indicating the FDR from SP assays was caused, in part, by toxicity and nonselective activity. To understand if the toxicity data from the SP assay itself could be used to predict toxic or nonspecific compounds, a third ROC analysis was performed on SP and DR toxicity data, revealing poor sensitivity when a cutoff of 25% nuclei reduction for putative toxicity was used (Fig. 4C). To measure the magnitude of how including nonselective compounds in DR confirmation affected the FDR and DR confirmation throughput, each toxic compound from the above-mentioned ROC calculations was assessed as either nonselective or selective despite being toxic at 10 μM (Fig. 4D). When analyzing putative hits for Prophylactic activity against schizonts, for example, we found that nine compounds exhibited toxicity at 10 μM in the DR assay, and of those, three exhibited a selectivity window and were likely true actives while six were either inactive or nonselective.

### Cross-comparisons of *P. vivax* liver stage chemosensitivity to other species and stages

**Figure 5:**
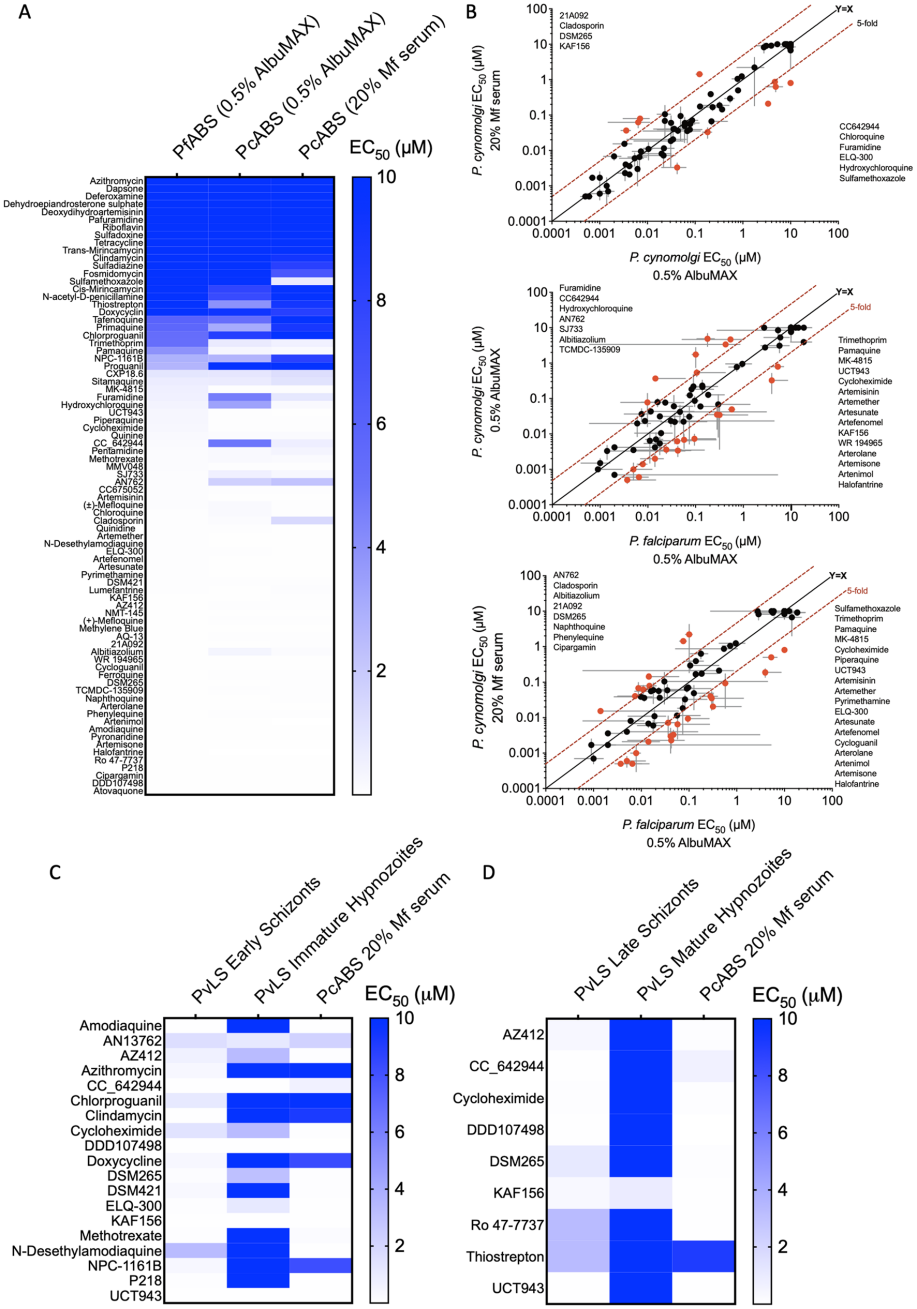
Screening the Reference Compound Library (RCL) against *P. cynomolgi* and *P. falciparum* asexual blood stages. (**A**) Potency heatmap of all 80 RCL compounds against *P. falciparum* asexual blood stages (PfABS) cultured in media supplemented with 0.5% Albumax versus *P. cynomolgi* asexual blood stages (PcABS) cultured in media supplemented with either 0.5% Albumax or 20% *M. fascicularis* (Mf) serum. (**B**) Breakout of data from ‘A,’ values indicate geometric mean of all independent experiments (two independent experiments for *P. cynomolgi* assays and at least two independent experiments for compounds found active in *P. falciparum* assays), red lines indicate a five-fold change in EC_50_ between the respective assays. Compounds with a > five-fold change are indicated with red circles and synonyms are listed in order least to most potent. Bars represent SD of all independent experiments. (**C**) Potency heatmap of RCL compounds with activity against PvLS in the Prophylactic assay mode versus PcABS. (**D**) Potency heatmap of RCL compounds with activity against PvLS in the Radical Cure assay mode versus PcABS. EC_50_ values at 10 µM, 3.33 µM, 1.11 µM denote EC_50_ of > 10 µM, EC_50_ of > 3.33 µM EC_50_ of > 1.11 µM respectively (KAF156 was inactive against mature hypnozoites at the maximum concentration tested of 1 µM).

To understand how the chemosensitivity of PvLS compares to other *Plasmodium* species and lifecycle stages, and to investigate if other *Plasmodium* screen platforms are predictive of hypnozonticidal activity, we first assayed the RCL against *P. cynomolgi* strain Berok K4 asexual blood stages (PcABS) and *P. falciparum* asexual blood stages (PfABS) to produce DR potency data (Fig. 5A). To better understand the effect of serum binding on each compound’s potency, the PcABS assay was performed in culture media containing two different serum supplements: one which is physiologically relevant and ideal for PcABS culture (20% *M. fascicularis* serum) and the other used in PfABS assays (0.5% Albumax, a bovine serum-replacement reagent)^34^. As shown in Fig. 5B, we found 10 compounds exhibited a five-fold shift in PcABS potency due to different serum conditions alone. When the RCL was assayed against both PcABS and PfABS in 0.5% Albumax to determine species specificity, 22 compounds exhibited five-fold shift in potency between the two species, which suggest differences in drug target or target conformation. When comparing potency against PcABS in 20% *M. fascicularis* serum versus PfABS in 0.5% Albumax, 26 compounds exhibited a five-fold difference in potency, of which 18 compounds exhibited a ten-fold increased potency against PcABS. Potency data from the RCL screen in both PcABS media conditions were then compared to potency data from the RCL screen against PvLS (Fig. 5C). Of the 19 compounds found active against early schizonts or immature hypnozoites in the PvLS Prophylactic assay, 14 were also active in the PcABS assay. Similarly, of the 9 compounds found active against late schizonts in the PvLS assay, 8 were active in the PcABS assay (Fig. 5D).

**Figure 6:**
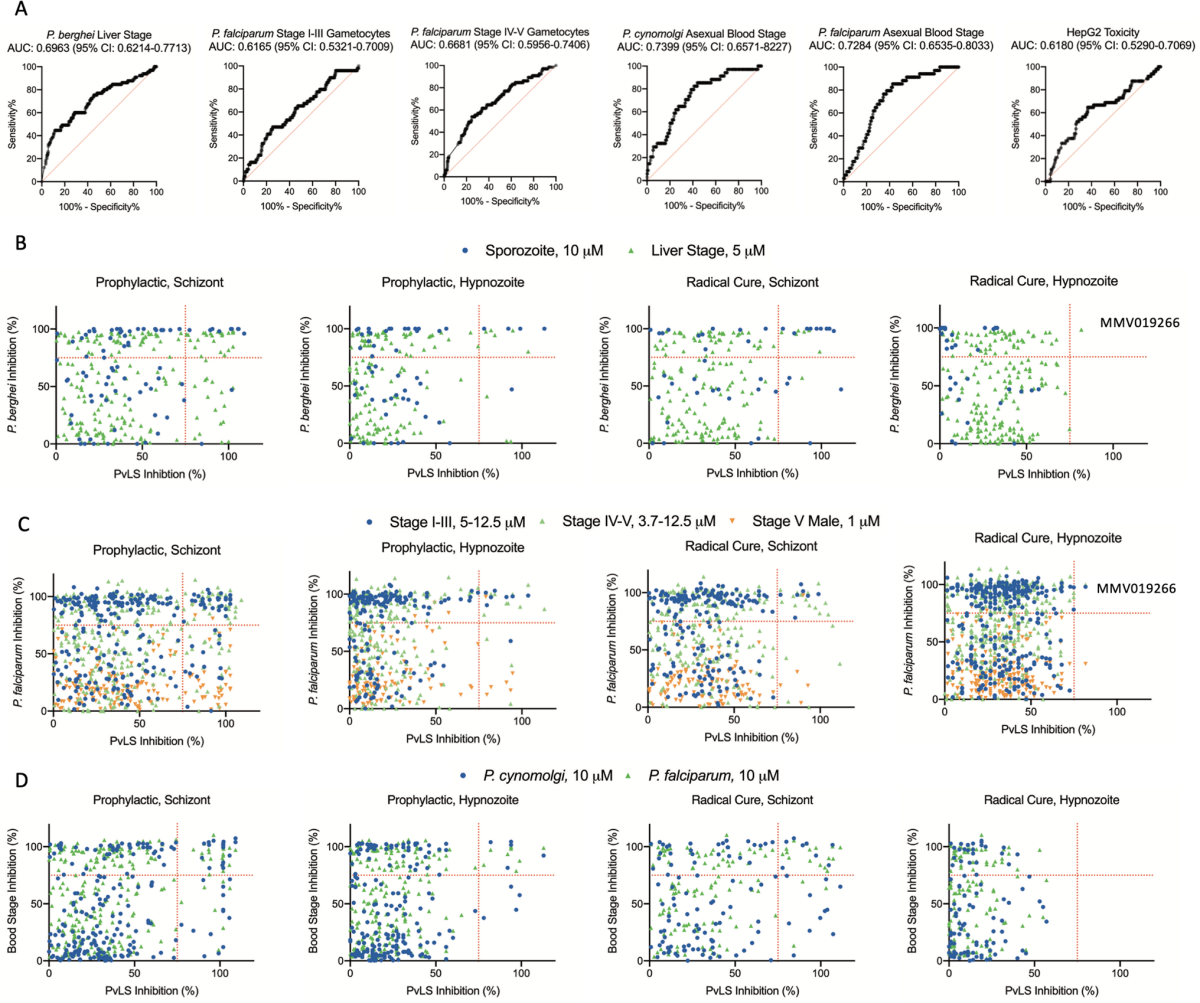
Cross-comparison of Single Point activity data from other *Plasmodium* species and lifecycle stages. (**A**) ROC curves showing predictive value of other datasets grouped by *P. berghei* liver stage, *P. falciparum* Stage I-III gametocytes, *P. falciparum* Stage IV–V gametocytes, *P. cynomolgi* asexual blood stage, *P. falciparum* asexual blood stage and hepatic toxicity. Non-vivax data was combined into a single number for each compound by using the highest inhibition value from the different assays which generated data for that species and stage; PvLS data was combined into a single number by using the highest inhibition value obtained against either hypnozoites or schizonts in either mode, and if any value was > 75%, that compound was deemed “active” (or, for hepatic toxicity, > 9% nuclei loss was deemed “toxic”). Area under curve (AUC) and 95% CI are indicated. (**B**–**D**) X axis is breakout of hypnozoite and schizont data by mode from ‘A,’ Y-axis is other species or stage activity data, Single Point test concentration is noted in legends. (**B**) Breakout of data *P. berghei* data from ‘A.’ (**C**) Breakout of *P. falciparum* gametocyte data from ‘A.’ (**D**) Breakout of *P. cynomolgi* and *P. falciparum* blood stage data from ‘A.’ Red lines indicate 75% activity cutoffs. MMV019266, a nonselective false positive, is noted in (**B**) and (**C**).

We next expanded the cross species and lifecycle stage comparisons by gathering SP activity data from three publications and the publicly-available Pathogen Box background data and aligning the activity data to our PvLS data by compound, thereby enabling side-by-side comparisons to assays against the *P. berghei* sporozoite and liver stage (PbLS)*,* the PcABS, the *P. falciparum* gametocyte stages, and the PfABS^26,27,34,80^. We first calculated additional ROC curves by simplifying the different PvLS endpoints as “active” if > 75% inhibition was achieved against either hypnozoites or schizonts in either mode and looked for predictive value from the other assays (Fig. 6A). Some predictive value was noted with the PcABS assay (AUC = 74%), the PfABS assays (AUC = 73%), and the PbLS assays (AUC = 70%), but none were as predictive as running the compounds through the PvLS SP assay itself (Fig. 4). To understand what sort of activity profile the other assays were predicting, the respective non-vivax assay data were compared to activity against hypnozoites or schizonts in Prophylactic or Radical Cure assays specifically, finding most of the enrichment was for early and late schizonticidal activity, with some enrichment for immature hypnozoite activity (Fig. 6B–D). To further confirm that compounds with activity in other SP *Plasmodium* assays are inactive against mature hypnozoites in the PvLS SP assay, and not a false negative in the PvLS SP assay, we collected and reviewed the DR curves for all runs of compounds for which we generated DR data and were previously reported to inhibit any other *Plasmodium* species or stage by 75% or greater in at least one SP assays (Fig. 6). Of the 51 compounds which met these criteria, we had performed at least one DR run for 10 compounds and more than one independent run for 41 others (Table S3). We found that, in every run of all 51 compounds, the DR data confirmed the compound’s inactivity against mature hypnozoites in the PvLS SP assay, indicating agreement between the PvLS SP and DR assays and a low false negative rate (> 97% specificity) for activity against mature hypnozoites in the PvLS SP assay. Lastly, because toxicity was found to contribute to FDR in our SP screen, we performed a ROC plot on HepG2 toxicity data from the PB^27^, finding almost no predictive value for the toxicity noted with primary hepatocytes in our assays (AUC = 62%, Fig. 6A).

## Discussion

From late 2016 to early 2020, over 20 Single Point (SP) and 58 dose response (DR) plate runs were performed at Shoklo Malaria Research Unit, Thailand, and Institute Pasteur of Cambodia, resulting in data for 1565 test compounds (Fig. 1A), including 1265 compounds tested for Prophylactic activity and 1465 compounds tested for Radical Cure activity in SP assays (Fig. 1B, C). At the start of screening, every compound and SP library was ran in both Prophylactic and Radical Cure modes, but after finding no new activity against mature hypnozoites in the RCL (Tables 1, 2) and publication that drug screening against *P. berghei* liver stage schizonts is capable of detecting Prophylactic activity against *P. vivax* schizonts^22^, we determined the assay’s sporozoite-limited throughput should be dedicated primarily to finding Radical Cure hits, especially against mature hypnozoites. Several elements of the assay were refined to provide better activity data and improve workflow (Figs. 2, S1B, S8, “Methods”).

Our Radical Cure-focused SP cascade resulted in no new hits against mature hypnozoites as DR assays alone were used to identify mature hypnozoite activity data for additional ionophores and three compounds (MMV018983, MMV021036 and MMV019204) first identified as active against *P. falciparum* stage V gametocytes. While MMV018983 and MMV019204 are not drug-like but possibly useful as chemical probes, MMV021036 is an aminothiazole and could be a suitable starting point for medicinal chemistry optimization^81^. Like MMV018983 and MMV019204, the additional ionophore hits could be used as chemical probes for investigating hypnozoite biology. Maduramicin, narasin, and salinomycin can all transport Na^+^ and have been hypothesized to kill *Plasmodium* and the related apicomplexan parasite *Toxoplasma *in vitro by disrupting ion gradients, especially proton gradients needed for proton exchangers, ultimately responsible for transporting Ca^2+^ into the acidocalcisome^82–84^. Similarly, nigericin is a H^+^ and K^+^ antiporter^85^ and also could cause deacidification of the acidocalcisome by directly disrupting intrahepatic or intraparasitic proton gradients. However, we have not attempted to confirm the presence of an acidocalcisome in hypnozoites nor have we confirmed the mechanism of action of ionophores against hypnozoites. As Ca^2+^ signaling has been shown to be essential for other critical lifecycle progressions in *Plasmodium*, if hypnozoites do possess an active acidocalcisome, this organelle could play a critical role in either maintaining or release from dormancy and would be an attractive target for biological and therapeutic research^86^*.*

Despite the lack of hypnozonticidal activity in the libraries we screened, interesting trends in schizonticidal activity were noted (Table 2). While most compounds with activity against late PvLS schizonts exhibited lesser potency than that exhibited when tested against other *Plasmodium* species and stages, three compounds exhibited more potency against late PvLS schizonts, indicating their mechanisms or targets could be distinctly important to the liver stage or *P. vivax* (Fig. S7). We found the PI4K inhibitor analog MMV085499^67^ potent against late PvLS schizonts (EC_50_ 1.2 nM) but was reported much less potent against *P. berghei* sporozoites (EC_50_ 280 nM) and the PfABS (EC_50_ in strains 3D7, Dd2, W2 ranged from 95 to 212 nM)^27^. This activity profile is unlike other PI4K inhibitors, such as MMV390048, which was relatively equipotent against the PfABS^87^ (EC_50_ 28 nM) and late PvLS schizonts (EC_50_ 7 nM, n = 40, Fig. 2E). Previously described as interfering with merozoite egress^69^, MMV026356 potently inhibited late PvLS schizonts (EC_50_ 26.6 nM) and completely eliminated schizont and hypnozoites in Prophylactic assays, but was reported much less potent against *P. berghei* sporozoites (EC_50_ 1.6 μM), the PfABS (EC_50_ in strains 3D7, Dd2, W2 ranged from 283 to 597 nM), and inactive against PcABS in a SP screen (57.5% inhibition)^27,34^. MMV023860 was modestly potent against late PvLS schizonts (EC_50_ 570 nM) but only micromolar potent against *P. berghei* sporozoites (EC_50_ 1.8 μM) and the PfABS (EC_50_ in strains 3D7, Dd2, W2 ranged from 1.5 to 1.9 μM), yet this compound was a top-tier hit against the PcABS (81.4% inhibition)^27,34^ suggesting the unknown target could have a distinct role in the relapsing clade of malaria species. Three compounds from the PB demonstrated complete inhibition of early schizonts and immature hypnozoites in a SP assay as well as submicromolar potency against late schizonts: MMV668727 (EC_50_ 190 nM), MMV675993 (EC_50_ 294 nM) and MMV026020 (EC_50_ 418 nM). MMV668727, a PI4K inhibitor analog, is characterized as active against onchocerciasis^27^ while MMV675993 is characterized as active against cryptosporidiosis^27^ but has also been reported to have activity against the pathogenic free-living amoeba *Balamuthia mandrillaris* (EC_50_ 6.35 μM)^88^, demonstrating interesting pan-pathogen activity among this set.

In both our previous report^19^ and this study we were unable to demonstrate 8AQ activity in an 8-day Radical Cure assay (Fig. S2A, B). The inactivity of primaquine could be due to several factors, including the metabolic state of the host hepatocytes, which activate primaquine via cytochrome 2D6^89^, or because three days of treatment in vitro does not reproduce the 7–14 day regimen used clinically for radical cure^90^. While the role of hepatic metabolism on the antirelapse efficacy of tafenoquine is unresolved^11,91^, three days of treatment in vitro should be sufficient as a single dose in coadministration with chloroquine in clinically effective^11^. Alternatively, inactivity in the 8-day assay format is likely due to false omission caused by the slow clearance of treated forms as we were able to show activity of 8AQs alone in vitro by modifying the assay to run for 20 days post-infection (Figs. 1B, 3). Interestingly, after 20 days we noted schizont signal in wells treated with lower, ineffective doses of 8AQ (with or without chloroquine), despite the removal of schizonts at day 9 and 10 post-infection using a PI4K inhibitor^18^ (Fig. 3). This signal can be explained by the presence of schizonts which arose from mature hypnozoites which were refractory to PI4Ki during treatment, this being further evidence of reactivation in vitro^16,37^. The 20-day schizont signal also provides another indication of activity: regardless of the viability of hypnozoites remaining in culture after 20 days, the absence of schizonts at higher doses suggests successful antirelapse activity. Together, these results provide in vitro confirmation of experimental and anecdotal evidence of synergy between 8AQs and chloroquine, which has yet to be demonstrated for *P. vivax*, and has only been recently been reported for primaquine-treated *P. cynomolgi*^76^. As chloroquine resistance is spreading geographically, this unique assay is an invaluable tool for assessing synergistic combinations prior to lengthy and expensive in vivo and clinical studies^10^. While we were able to run three independent experiments of our 20-day assay to assess 8AQ activity, running such an assay routinely for all PvLS screening is not practical as it requires more sporozoites per plate and is subject to increase risk of loss due to contamination from mosquito microbiota delivered during sporozoite infection. Rather, this assay could also be used in a screening cascade after DR confirmation of hits to characterize the potency of antirelapse compounds under consideration for, or already in, drug development.

Despite a high FDR across endpoints, analyses of screen data reveals the SP assay suitably predicted true activity against early and late schizonts as well as immature hypnozoites (Fig. 4). Additionally, the activity of ionophore controls against mature hypnozoites was consistently detected in SP assays (Fig. S3D). We found toxicity was a frequently-confounding factor lending to the FDR. As an example, the MB compound MMV019266 was disappointing in that it was active against late PvLS schizonts (EC_50_ 1.62 μM) and most other extra-hepatic stages of the *Plasmodium* lifecycle^26^ and was originally observed to have activity against mature hypnozoites when tested in the SP screen at 10 μM, but in DR confirmation was found to be slightly toxic above 16.7 μM and therefore was not a true positive (Table S2, Fig. 6B, C). While the FDR could be reduced if toxic compounds were pre-screened out of the cascade, we found including toxic hits in DR confirmation would only minimally impact throughput and prevent an increase in FOR due to the possibility of compounds which are toxic at the SP dose but also selective against PvLS (Fig. 4D). The nonexistent hit rate for activity against mature hypnozoites in SP assays and remarkably low hit rate for mature hypnozoite activity overall, just three of 1561 non-ionophores (0.19%), was surprising in that many of the libraries we tested were either malaria or other disease-focused and were therefore curated to be enriched for presumably desirable attributes such as *Plasmodium* blood stage activity^28^. As an example, while many hits were noted in Prophylactic assays (Figs. S5, S6), the MB and SB were almost devoid of activity in Radical Cure assays as only one compound with sub-micromolar activity against late schizonts was found in each library: MMV690491 (EC_50_ 395 nM) and MMV396797 (EC_50_ 599 nM, Table 2). To investigate this discrepancy, we generated potency data for the RCL against the PcABS and found a high level of agreement between the PfABS, PcABS and PvLS assays for schizonticidal compounds but only confirmation of inactivity of schizonticidal compound against mature hypnozoites (Fig. 5). Likewise, an expanded comparison of our SP PvLS activity data to published SP activity data against multiple *Plasmodium* stages and species found other *Plasmodium* assays can predict, with some degree of overlap, activity against early hypnozoites, early schizonts, and late schizonts, but no hits from any other *Plasmodium* assay were active against mature hypnozoites (Fig. 6B–D). While this result should be considered with the caveats that SP data can be statistically noisy and based on multiple dependent variables (i.e. assay time, SP dose used, activity cutoff, and endpoint method), it does suggest that alternative assays such as the PbLS, PcABS, and PfABS assays can be used in a screen cascade for PvLS schizonticidal or Prophylactic activity, but not Radical Cure activity. Rather, this result is indicative of the unique insensitivity of mature hypnozoites and suggests adding one or more of these other assays as a pre-screen in our cascade would likely not enrich for mature hypnozoite hits and could, in fact, be detrimental by contributing to FOR. Future studies include the pursuit of additional mature hypnozoite hits by screening of larger, more diverse chemical libraries, made possible by the advances and information gained in this report.

## Methods

### Library preparation

Small volume 384-well plates (Greiner part 784261) were spotted with 5 μL of each test and control compound, resulting in a 1000× concentration source plate for a 40 nL pin tool (Fig. S1A). For SP assays, libraries were provided preplated at 10 mM in DMSO (MMV). Control compounds primaquine and monensin were plated at 10 mM, MMV390048 (PI4Ki) and atovaquone were plated at 3.3 mM, and nigericin was plated at 200 μM. For DR assays, serial dilutions were made in DMSO (100%) using a Biomek 4000 (Beckman Coulter). Two DR plate maps were used in this study, termed v1 as originally published^19^ and v2 (Fig. S1B). The assay steps were unchanged regardless of the map version used. Source plates were sealed and shipped on dry ice to Shoklo Malaria Research Unit in Mae Sot, Thailand or Institute Pasteur in Phnom Penh, Cambodia.

### Experimental mosquito infections and ethical consideration

Mosquitoes were reared and infected with a vivax malaria patient isolate bloodmeal to enable harvesting of sporozoites as previously described^19^. The Thai human subjects protocols for this study were approved by the Institutional Ethics Committee of the Thai Ministry of Public Health and the Oxford Tropical Medicine Ethical Committee (TMEC 14-016 and OxTREC 40-14). The Cambodian human subjects protocols for this study were approved by the Cambodian National Ethics Committee for Health Research (100NECHR, 111NECHR, and 113NHECR). Protocols conformed to the Helsinki Declaration on ethical principles for medical research involving human subjects (version 2002) and informed written consent was obtained from all volunteers or legal guardians.

### Hepatocyte culture, infection, treatment, and immunofluorescent staining

Three different lots of primary human hepatocytes (BioIVT) were validated for PvLS assays and used in this study: PDC were used first, followed by UBV, and then BGW. Assay plates were seeded, infected, treated, and stained as previously described^19^ with the one modification: media change was performed by placing the assay plate, inverted, into a custom-fabricated aluminum holder and spinning at 40 RCF for 30 s with the slowest possible acceleration. Plates were then moved into a sterile field, excess spent media was removed by blotting with an autoclaved, lint-free cloth (VWR part 100488-446), and 40 μL Plate media (BioIVT part Z99029, with 10% bovine sera) was added per well.

### Twelve and twenty-day 8-aminoquinoline assay

Cultures were seeded with primary hepatocytes, infected with sporozoites, and treated following the Radical Cure mode. At days 9 and 10 post-infection 1 μM PI4Ki MMV390048 (MMV) was added to the culture media. Because of added PvLS parasite growth variability per well from such a long-term assay, the number of points in the dose response was cut to 6 (50 μM–48 nM in a 1:4 dilution series) to increase replicates per concentration (Fig. S1B). For each of the three independent experiments, two assay plates were seeded with hepatocytes, infected with sporozoites, and treated with compound together. One assay plate was then fixed at 12 days post-infection, and the other at 20 days post-infection. Assay plates were then stained, imaged, and quantified as described below.

### High content imaging, and analysis

Initially, cold-chain shipments were used to return completed assay plates to UGA for high content imaging (HCI) with an ImageXpress (IMX) Micro confocal microscope (Molecular Devices). Assay turnaround was dramatically shortened by the installation of a Lionheart FX imager (Biotek) at IPC. This system was found capable of imaging an entire 384-well plate (4 images per well per channel) in less than 4 h. Data was either analyzed at IPC or compressed and uploaded to cloud storage for further analysis. We found a 4× objective on the Lionheart provided sufficient image resolution to obtain HCI data highly correlating to that obtained with a 10× objective on the IMX while also reducing imaging time and file size (Fig. S8). Using built-in software (MetaXpress for the IMX and Gen5 for the Lionheart), parasites were masked and quantified for growth area per well. Hypnozoites were defined as parasites having a minimum area of 35 μm^2^ and maximum area of 150–180 μm^2^, while schizonts were found to have > 500 μm^2^ area^36^. Hepatic nuclei were also quantified for every well of every plate and used to measure compound toxicity.

### Normalization, putative hit selection, and curve fitting

CDD Vault was used for compound management, plate mapping, normalization, data storage, and visibility. For v1 DR assays, PI4Ki was used as the positive control for normalization of schizonts and hypnozoites in Prophylactic assays as well as schizonts in Radical Cure assays. Monensin was not yet known to have an effect on hypnozoites in Radical Cure assays so data were normalized to DMSO control wells and inhibition was calculated as 100%—decrease in hypnozoite quantity. For v2 DR assays, we first used monensin and then nigericin as the positive control for hypnozoites and schizonts in both Prophylactic and Radical Cure assays (Fig. 2E). Similarly, the RCL, SB, and PB were all plated and assayed prior to regular use of ionophore controls, thus PI4Ki was used and normalization performed the same as for DR assays describe above. Later, the MB and PD were plated and assayed with monensin control wells (Fig. S3A). Because, prior to the availability of HCI at IPC, imaging and quality assessment of individual SP runs was dependent on shipping and could not be performed until long after runs were complete, several libraries were run twice prior to obtaining results in order to improve the likelihood of obtaining a high-quality run from a single round of shipping. Therefore, multiple SP runs of several libraries were available for comparative analysis (Fig. S3A, C, D). Schizont and hypnozoite inhibition from two runs of the RCL were compared and a cluster of putative hits was found in both the superior and inferior run at > 75% inhibition, thus this became the cutoff for putative hit confirmation in a DR assay (Fig. S3C). As previously described^36^, we noted treated parasite clearance was diminished at toxic doses of compound, thus points on DR curves in which toxicity and a bell-shape curve for parasite inhibition were noted were removed for curve fitting (Fig. 2B, C). An EC_50_ value for parasite growth (or CC_20_ value for hepatic toxicity) could not be calculated for most curves with shallow slopes, only one point demonstrating > 50% inhibition, or with no top plateau, thus a curve was characterized as > ([maximum] with < 50% activity) when activity was apparent. Some compound activity resulted in a non-sigmoidal, but possibly active, curve which was characterized as “ND” for “EC_50_ not determined” (Tables 1, 2, Tables S1, S2).

### Receiver-operate characteristic analyses

Receiver-operate characteristic (ROC) curves were calculated by comparing SP and DR activity for all compounds for which both types of data existed. The SP data for ROC analysis were collected from the highest-quality run of SP libraries. When a compound was represented in more than one library, the replicate SP data from each library was kept for downstream analysis. However, for control compounds which were also represented as a test well in the library itself, only the data specifically from the test well (not the control wells) was kept for downstream analysis. The activity of every compound in the full DR dataset was then characterized by separately reviewing and describing each DR curve as inactive, active, or ambiguous. A curve was determined inactive when there was little or no activity at 10 μM (the SP dose used), regardless of whether or not the compound appeared active at higher doses when tested from 50 μM in the DR assay, while a curve was determined “active” when there was a full or partial inhibition (> 60%) or sigmoidal fitting. A curve was determined ambiguous when the activity was likely not due to assay noise but also not sigmoidal (indicating likely nonselective activity), when the maximal inhibition at the highest dose was likely true activity but also not resulting in more than 50–60% inhibition, or when the inflection point of an active curve was directly at or near (less than 1 dilution from) 10 μM. Finally, the characterized all-inclusive DR dataset was merged with the all-inclusive SP dataset described above and analyzed using the ROC calculation tool in Graphpad Prism.

### Cross species and stage comparisons

The RCL was tested against PcABS by serially diluting the 5 mM compound stock three-fold with DMSO (100%) to generate a 10-point DR. A 500-fold dilution (100 nL) of compound from the master plate were spotted onto a 384-well assay plate using the Echo 550 liquid dispenser (Labcyte). The plates were then sealed with a removable foil seal using a PlateLoc Thermal Microplate Sealer (Agilent) until use. Mefloquine (5 mM) and DMSO (100%) were used within the master compound plates as the background and baseline controls respectively. The PcABS SYBRI proliferation assay was performed as previously described^34^. Potency data for the RCL against the PfABS was obtained by running a 72-h Sybr green assay with strain 3D7 as previously described^92^.

Activity data for the *P. berghei* liver stage, *P. falciparum* gametocytes, the *P. cynomolgi* blood stage, the *P. falciparum* blood stage, and HepG2 toxicity data were extracted from the respective publications, loaded into CDD Vault by compound, and exported alongside our PvLS data. Because data was available from multiple different assays against a specific species and stage, to manage the number of comparisons ROC analyses were performed by using the maximum inhibition value from any assay for the respective species and stage combination with two exceptions: in one assay the Malaria Box was tested at 50 μM against the *P. berghei* liver stage and resulted in inhibition and toxicity for most compounds tested, indicating an excessive dose was used, and Female stage V gametocytes appeared refractory to all Malaria Box compounds, thus only Male data were plotted^26^. Likewise, the maximum inhibition observed, against either hypnozoites or schizonts in either mode, was used as a single value for PvLS data with 75% inhibition serving as the cutoff for “active”.

### Figure generation and statistics

Venn diagrams of library size and overlap were created using Meta-Chart (https://www.meta-chart.com/venn). Graphpad Prism was used to generate publication-quality charts and calculate EC_50_ values when displaying multiple independent experiments. CDD Vault was used to fit DR curves and calculate EC_50_ values from single independent experiments (Tables 1, 2, Tables S1, S2). At least two independent experiments were used to confirm the relative potency of all active compounds in Tables 1, 2, S1, and S2 except lasalocid A, cycloheximide, MMV690491, and MMV690648 in Tables 2 and S2, for which only one DR run was obtained. Due to logistical considerations, the screening cascade and assay refinement occurred concurrently, thus some of the DR curves described herein are from v1 assays. Because data for each compound was collected from running a specific synthetic batch in one of three lots of primary human hepatocytes infected with *P. vivax* parasites from a unique patient isolate in two different countries, averaging multiple runs was determined to represent too many variables and not properly describe the data, so the run with the highest run quality is shown. Error bars and independent experimental replication are described in respective figure legends. Coefficient of variance (CV) was calculated by 100*(Standard Deviation/Average). Z′ factor was calculated as previously described, using DMSO control wells as negative control and ionophore control wells as positive control^78^.

## Supplementary Information

Supplementary Information 1.

Supplementary Information 2.

Supplementary Information 3.

Supplementary Information 4.

Supplementary Information 5.

Supplementary Information 6.

## Data Availability

Most of the data generated during this study are included in this published article and its Supplementary Information files. High content imaging files generated and analyzed during this study are available from the corresponding author on reasonable request.
