# Supplementary material for: Probing the distinct chemosensitivity of Plasmodium vivax liver stage parasites and demonstration of 8-aminoquinoline radical cure activity in vitro
Source: Sci Rep. 2021 Oct 7;11:19905. doi: 10.1038/s41598-021-99152-9 (PMC8497498; doi:10.1038/s41598-021-99152-9)
Supplement: Supplementary file 1 — Supplementary Information 1. [file 41598_2021_99152_MOESM1_ESM.pdf]

Supplemental Materials

Title
Probing the distinct chemosensitivity of *Plasmodium vivax* liver stage parasites and demonstration of 8-aminoquinoline radical cure activity *in vitro*

Steven P. Maher[1†]*, Amélie Vantaux[2†], Victor Chaumeau[3,4], Adeline C. Y. Chua[5,6,7], Caitlin A. Cooper[1], Chiara Andolina[3,4], Julie Péneau[2], Mélanie Rouillier[8], Zaira Rizopoulos[8], Sivchheng Phal[2], Eakpor Piv[2], Chantrea Vong[2], Sreyvouch Phen[2], Chansophea Chhin[2], Baura Tat[2], Sivkeng Ouk[2], Bros Doeurk[2], Saorin Kim[2], Sangrawee Suriyakan[3], Praphan Kittiphanakun[3], Nana Akua Awuku[1], Amy J. Conway[9], Rays H.Y. Jiang[9], Bruce Russell[7], Pablo Bifani[5,6,10,11], Brice Campo[8], François Nosten[3, 4], Benoît Witkowski[2]*, Dennis Kyle[1]*

Contents

Supplementary Figure 1
Supplementary Figure 2
Supplementary Figure 3
Supplementary Figure 4
Supplementary Figure 5
Supplementary Figure 6
Supplementary Figure 7
Supplementary Figure 8
Supplemental Table 1 (Excel file): All Prophylactic (6-day assay) Actives
Supplemental Table 2 (Excel file): All Radical Cure (8-day assay) Actives
Supplemental Table 3 (Excel file): All Radical Cure (8-day assay) Hypnozoite DR Curves, when performed, for compounds with >75% inhibition in other SP *Plasmodium* Assays
Supplemental Data File: tabs include raw data for all main and supplemental figures

A

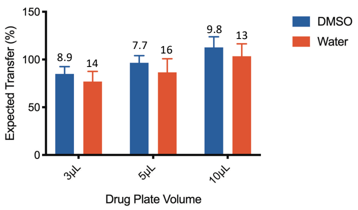

B

v1 Assay Plate Layout																									
		Test Compounds 1-12								Test Compounds 13-24								Test Compounds 25-36							
		1	2	3	4	5	6	7	8	9	10	11	12	13	14	15	16	17	18	19	20	21	22	23	24
Atovaquone	A	3300.00	1100.00	366.67	122.22	40.74	13.58	4.53	1.51	3300.00	1100.00	366.67	122.22	40.74	13.58	4.53	1.51	3300.00	1100.00	366.67	122.22	40.74	13.58	4.53	1.51
PI4Ki (MMV390048)	B	3300.00	1100.00	366.67	122.22	40.74	13.58	4.53	1.51	3300.00	1100.00	366.67	122.22	40.74	13.58	4.53	1.51	3300.00	1100.00	366.67	122.22	40.74	13.58	4.53	1.51
Monensin or Nigericin	C	200.00	66.67	22.22	7.41	2.47	0.82	0.27	0.09	200.00	66.67	22.22	7.41	2.47	0.82	0.27	0.09	200.00	66.67	22.22	7.41	2.47	0.82	0.27	0.09
Test Compuond	D	10000.00	3333.33	1111.11	370.37	123.46	41.15	13.72	4.57	10000.00	3333.33	1111.11	370.37	123.46	41.15	13.72	4.57	10000.00	3333.33	1111.11	370.37	123.46	41.15	13.72	4.57
Test Compuond	E	10000.00	3333.33	1111.11	370.37	123.46	41.15	13.72	4.57	10000.00	3333.33	1111.11	370.37	123.46	41.15	13.72	4.57	10000.00	3333.33	1111.11	370.37	123.46	41.15	13.72	4.57
Test Compuond	F	10000.00	3333.33	1111.11	370.37	123.46	41.15	13.72	4.57	10000.00	3333.33	1111.11	370.37	123.46	41.15	13.72	4.57	10000.00	3333.33	1111.11	370.37	123.46	41.15	13.72	4.57
Test Compuond	G	10000.00	3333.33	1111.11	370.37	123.46	41.15	13.72	4.57	10000.00	3333.33	1111.11	370.37	123.46	41.15	13.72	4.57	10000.00	3333.33	1111.11	370.37	123.46	41.15	13.72	4.57
Test Compuond	H	10000.00	3333.33	1111.11	370.37	123.46	41.15	13.72	4.57	10000.00	3333.33	1111.11	370.37	123.46	41.15	13.72	4.57	10000.00	3333.33	1111.11	370.37	123.46	41.15	13.72	4.57
Test Compuond	I	10000.00	3333.33	1111.11	370.37	123.46	41.15	13.72	4.57	10000.00	3333.33	1111.11	370.37	123.46	41.15	13.72	4.57	10000.00	3333.33	1111.11	370.37	123.46	41.15	13.72	4.57
Test Compuond	J	10000.00	3333.33	1111.11	370.37	123.46	41.15	13.72	4.57	10000.00	3333.33	1111.11	370.37	123.46	41.15	13.72	4.57	10000.00	3333.33	1111.11	370.37	123.46	41.15	13.72	4.57
Test Compuond	K	10000.00	3333.33	1111.11	370.37	123.46	41.15	13.72	4.57	10000.00	3333.33	1111.11	370.37	123.46	41.15	13.72	4.57	10000.00	3333.33	1111.11	370.37	123.46	41.15	13.72	4.57
Test Compuond	L	10000.00	3333.33	1111.11	370.37	123.46	41.15	13.72	4.57	10000.00	3333.33	1111.11	370.37	123.46	41.15	13.72	4.57	10000.00	3333.33	1111.11	370.37	123.46	41.15	13.72	4.57
Test Compuond	M	10000.00	3333.33	1111.11	370.37	123.46	41.15	13.72	4.57	10000.00	3333.33	1111.11	370.37	123.46	41.15	13.72	4.57	10000.00	3333.33	1111.11	370.37	123.46	41.15	13.72	4.57
Test Compuond	N	10000.00	3333.33	1111.11	370.37	123.46	41.15	13.72	4.57	10000.00	3333.33	1111.11	370.37	123.46	41.15	13.72	4.57	10000.00	3333.33	1111.11	370.37	123.46	41.15	13.72	4.57
Test Compuond	O	10000.00	3333.33	1111.11	370.37	123.46	41.15	13.72	4.57	10000.00	3333.33	1111.11	370.37	123.46	41.15	13.72	4.57	10000.00	3333.33	1111.11	370.37	123.46	41.15	13.72	4.57
DMSO	P	DMSO	DMSO	DMSO	DMSO	DMSO	DMSO	DMSO	DMSO	DMSO	DMSO	DMSO	DMSO	DMSO	DMSO	DMSO	DMSO	DMSO	DMSO	DMSO	DMSO	DMSO	DMSO	DMSO	DMSO

v2 Assay Plate Layout																									
		1	2	3	4	5	6	7	8	9	10	11	12	13	14	15	16	17	18	19	20	21	22	23	24
Atovaquone	A	3300.00	1100.00	366.67	122.22	40.74	13.58	4.53	1.51	0.50	0.17	0.06	0.02	3300.00	1100.00	366.67	122.22	40.74	13.58	4.53	1.51	0.50	0.17	0.06	0.02
PI4Ki (MMV390048)	B	3300.00	1100.00	366.67	122.22	40.74	13.58	4.53	1.51	0.50	0.17	0.06	0.02	3300.00	1100.00	366.67	122.22	40.74	13.58	4.53	1.51	0.50	0.17	0.06	0.02
Monensin or Nigericin	C	200.00	66.67	22.22	7.41	2.47	0.82	0.27	0.09	0.03	0.01	0.00	0.00	200.00	66.67	22.22	7.41	2.47	0.82	0.27	0.09	0.03	0.01	0.00	0.00
Test Compound 1	D	50000.00	16666.67	5555.56	1851.85	617.28	205.76	68.59	22.86	7.62	2.54	0.85	0.28	50000.00	16666.67	5555.56	1851.85	617.28	205.76	68.59	22.86	7.62	2.54	0.85	0.28
Test Compound 2	E	50000.00	16666.67	5555.56	1851.85	617.28	205.76	68.59	22.86	7.62	2.54	0.85	0.28	50000.00	16666.67	5555.56	1851.85	617.28	205.76	68.59	22.86	7.62	2.54	0.85	0.28
Test Compound 3	F	50000.00	16666.67	5555.56	1851.85	617.28	205.76	68.59	22.86	7.62	2.54	0.85	0.28	50000.00	16666.67	5555.56	1851.85	617.28	205.76	68.59	22.86	7.62	2.54	0.85	0.28
Test Compound 4	G	50000.00	16666.67	5555.56	1851.85	617.28	205.76	68.59	22.86	7.62	2.54	0.85	0.28	50000.00	16666.67	5555.56	1851.85	617.28	205.76	68.59	22.86	7.62	2.54	0.85	0.28
Test Compound 5	H	50000.00	16666.67	5555.56	1851.85	617.28	205.76	68.59	22.86	7.62	2.54	0.85	0.28	50000.00	16666.67	5555.56	1851.85	617.28	205.76	68.59	22.86	7.62	2.54	0.85	0.28
Test Compound 6	I	50000.00	16666.67	5555.56	1851.85	617.28	205.76	68.59	22.86	7.62	2.54	0.85	0.28	50000.00	16666.67	5555.56	1851.85	617.28	205.76	68.59	22.86	7.62	2.54	0.85	0.28
Test Compound 7	J	50000.00	16666.67	5555.56	1851.85	617.28	205.76	68.59	22.86	7.62	2.54	0.85	0.28	50000.00	16666.67	5555.56	1851.85	617.28	205.76	68.59	22.86	7.62	2.54	0.85	0.28
Test Compound 8	K	50000.00	16666.67	5555.56	1851.85	617.28	205.76	68.59	22.86	7.62	2.54	0.85	0.28	50000.00	16666.67	5555.56	1851.85	617.28	205.76	68.59	22.86	7.62	2.54	0.85	0.28
Test Compound 9	L	50000.00	16666.67	5555.56	1851.85	617.28	205.76	68.59	22.86	7.62	2.54	0.85	0.28	50000.00	16666.67	5555.56	1851.85	617.28	205.76	68.59	22.86	7.62	2.54	0.85	0.28
Test Compound 10	M	50000.00	16666.67	5555.56	1851.85	617.28	205.76	68.59	22.86	7.62	2.54	0.85	0.28	50000.00	16666.67	5555.56	1851.85	617.28	205.76	68.59	22.86	7.62	2.54	0.85	0.28
Test Compound 11	N	50000.00	16666.67	5555.56	1851.85	617.28	205.76	68.59	22.86	7.62	2.54	0.85	0.28	50000.00	16666.67	5555.56	1851.85	617.28	205.76	68.59	22.86	7.62	2.54	0.85	0.28
Test Compound 12	O	50000.00	16666.67	5555.56	1851.85	617.28	205.76	68.59	22.86	7.62	2.54	0.85	0.28	50000.00	16666.67	5555.56	1851.85	617.28	205.76	68.59	22.86	7.62	2.54	0.85	0.28
DMSO	P	DMSO	DMSO	DMSO	DMSO	DMSO	DMSO	DMSO	DMSO	DMSO	DMSO	DMSO	DMSO	DMSO	DMSO	DMSO	DMSO	DMSO	DMSO	DMSO	DMSO	DMSO	DMSO	DMSO	DMSO

20-day Assay Plate Layout																									
		1	2	3	4	5	6	7	8	9	10	11	12	13	14	15	16	17	18	19	20	21	22	23	24
Atovaquone	A	3300.00	825.00	206.25	51.56	12.89	3.22	3300.00	825.00	206.25	51.56	12.89	3.22	3300.00	825.00	206.25	51.56	12.89	3.22	3300.00	825.00	206.25	51.56	12.89	3.22
PI4Ki (MMV390048)	B	3300.00	825.00	206.25	51.56	12.89	3.22	3300.00	825.00	206.25	51.56	12.89	3.22	3300.00	825.00	206.25	51.56	12.89	3.22	3300.00	825.00	206.25	51.56	12.89	3.22
Nigericin	C	200.00	50.00	12.50	3.13	0.78	0.20	200.00	50.00	12.50	3.13	0.78	0.20	200.00	50.00	12.50	3.13	0.78	0.20	200.00	50.00	12.50	3.13	0.78	0.20
Test Compound 1	D	50000.00	12500.00	3125.00	781.25	195.31	48.83	50000.00	12500.00	3125.00	781.25	195.31	48.83	50000.00	12500.00	3125.00	781.25	195.31	48.83	50000.00	12500.00	3125.00	781.25	195.31	48.83
Test Compound 2	E	50000.00	12500.00	3125.00	781.25	195.31	48.83	50000.00	12500.00	3125.00	781.25	195.31	48.83	50000.00	12500.00	3125.00	781.25	195.31	48.83	50000.00	12500.00	3125.00	781.25	195.31	48.83
Test Compound 3	F	50000.00	12500.00	3125.00	781.25	195.31	48.83	50000.00	12500.00	3125.00	781.25	195.31	48.83	50000.00	12500.00	3125.00	781.25	195.31	48.83	50000.00	12500.00	3125.00	781.25	195.31	48.83
Test Compound 4	G	50000.00	12500.00	3125.00	781.25	195.31	48.83	50000.00	12500.00	3125.00	781.25	195.31	48.83	50000.00	12500.00	3125.00	781.25	195.31	48.83	50000.00	12500.00	3125.00	781.25	195.31	48.83
Test Compound 5	H	50000.00	12500.00	3125.00	781.25	195.31	48.83	50000.00	12500.00	3125.00	781.25	195.31	48.83	50000.00	12500.00	3125.00	781.25	195.31	48.83	50000.00	12500.00	3125.00	781.25	195.31	48.83
Test Compound 6	I	50000.00	12500.00	3125.00	781.25	195.31	48.83	50000.00	12500.00	3125.00	781.25	195.31	48.83	50000.00	12500.00	3125.00	781.25	195.31	48.83	50000.00	12500.00	3125.00	781.25	195.31	48.83
Test Compound 7	J	50000.00	12500.00	3125.00	781.25	195.31	48.83	50000.00	12500.00	3125.00	781.25	195.31	48.83	50000.00	12500.00	3125.00	781.25	195.31	48.83	50000.00	12500.00	3125.00	781.25	195.31	48.83
Test Compound 8	K	50000.00	12500.00	3125.00	781.25	195.31	48.83	50000.00	12500.00	3125.00	781.25	195.31	48.83	50000.00	12500.00	3125.00	781.25	195.31	48.83	50000.00	12500.00	3125.00	781.25	195.31	48.83
Test Compound 9	L	50000.00	12500.00	3125.00	781.25	195.31	48.83	50000.00	12500.00	3125.00	781.25	195.31	48.83	50000.00	12500.00	3125.00	781.25	195.31	48.83	50000.00	12500.00	3125.00	781.25	195.31	48.83
Test Compound 10	M	50000.00	12500.00	3125.00	781.25	195.31	48.83	50000.00	12500.00	3125.00	781.25	195.31	48.83	50000.00	12500.00	3125.00	781.25	195.31	48.83	50000.00	12500.00	3125.00	781.25	195.31	48.83
Test Compound 11	N	50000.00	12500.00	3125.00	781.25	195.31	48.83	50000.00	12500.00	3125.00	781.25	195.31	48.83	50000.00	12500.00	3125.00	781.25	195.31	48.83	50000.00	12500.00	3125.00	781.25	195.31	48.83
Test Compound 12	O	50000.00	12500.00	3125.00	781.25	195.31	48.83	50000.00	12500.00	3125.00	781.25	195.31	48.83	50000.00	12500.00	3125.00	781.25	195.31	48.83	50000.00	12500.00	3125.00	781.25	195.31	48.83
DMSO	P	DMSO	DMSO	DMSO	DMSO	DMSO	DMSO	DMSO	DMSO	DMSO	DMSO	DMSO	DMSO	DMSO	DMSO	DMSO	DMSO	DMSO	DMSO	DMSO	DMSO	DMSO	DMSO	DMSO	DMSO

Figure S1. Methods and Assay Refinement. A) Fluorescein dye was diluted in water or DMSO and plated at 3, 5, or 10 µL per well prior to transfer by a 40 nL, hand-actuated pin tool into a 384-well assay plate with 40 µL culture media. Fluorescence intensity was measured to assess actual transfer versus expected transfer. Bars represent SD of 10 wells per condition, data are representative of two independent experiments. The CV (%) across wells for each condition is listed above respective bars. B) Plate maps for v1, v2, and 20-day formats, values represent µM in 1000x source plate and nM in assay plate.

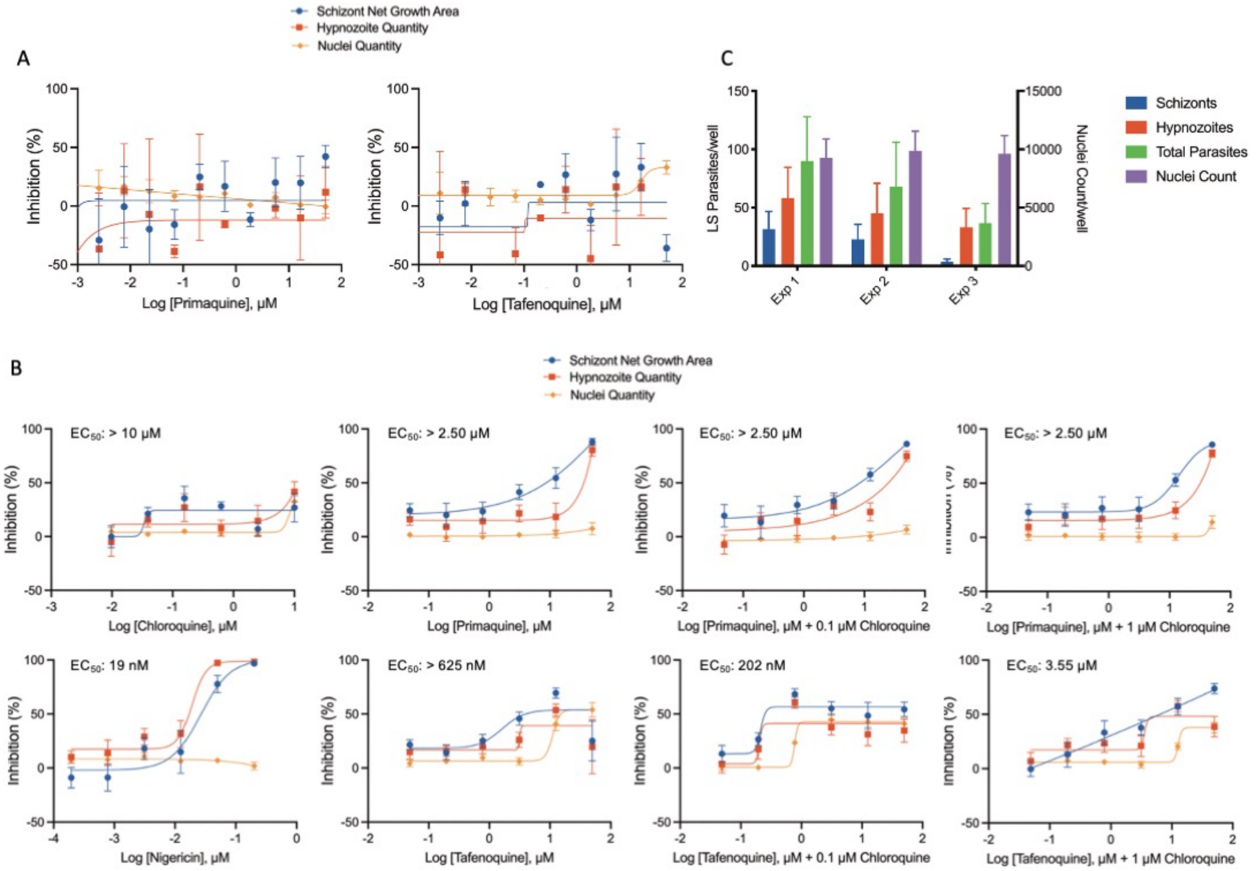

Figure S2. Potency of 8AQs with and without CQ in 8-day and 12-day Radical Cure assays and parasite infection numbers from DMSO control wells from the 20-day assay. A) Primaquine and tafenoquine were assayed in the v2 8-day Radical Cure assay format and found inactive. B) Chloroquine, primaquine and tafenoquine were assayed in a 12-day Radical Cure assay format alone and in combination with chloroquine as indicated on the X-axis. Chloroquine was found slightly toxic at 10 µM, thus the 0.1 and 1 µM combinations with 8AQs are shown. Nigericin was ran in all assays as a positive and normalization control. EC_{50} shown indicates potency against hypnozoites. Bars represent the SEM of three independent experiments; the first two experiments were the average of two replicate wells per concentration, the third experiment was the average of four replicate wells for each concentration. C) High Content Imaging quantification of hypnozoites, schizonts, total PvLS parasites, and hepatic nuclei per DMSO negative control wells for each of the three independent 20-day experiments. Bars represent SD.

A

Library	DMSO wells (n)	Positive Control wells (n)	Positive Control	Mode	Run	Sch/well (SD)	Hyp/well (SD)	CV Sch	CV Hyp	Z' Sch	Z' Hyp	Z Sch	Z Hyp
Reference Compound Library	15	6	PI4Ki	Prophylactic	Superior	24 (±7.7)	62 (±6.3)	32	20	-0.28	0.13	-1.7	-0.18
					Inferior	11 (±2.0)	33 (±5.1)	35	31	-0.33	-0.11	-2.6	-1.09
				Radical Cure	n/a	17 (±2.9)	38 (±3.8)	33	20	-1.07	n/a	-1.48	n/a
Pathogen and Stasis Boxes	20	20	PI4Ki	Prophylactic	n/a	16 (±2.5)	82 (±5.6)	32	13.5	0.13	0.27	-0.52	-0.18
				Radical Cure	n/a	31 (±2.4)	38 (±3.0)	15	16	0.04	n/a	-0.65	n/a
Malaria Box	19	8	Monensin	Prophylactic	Superior	18 (±2.6)	153 (±14)	30	19	0.07	0.47	-0.97	0.11
					Inferior	7.2 (±1.8)	22 (±2.5)	49	23	-1.3	-0.51	-2.57	-0.8
				Radical Cure	Superior	14 (±1.8)	43 (±3.4)	26	16	-0.21	0.45	-0.39	-0.25
					Inferior	12 (±2.2)	26 (±5.2)	38	40	-0.68	-1.01	-2.86	-0.91
Photodiversity	12	6	Monensin	Radical Cure	Superior	20 (±2.8)	41 (±4.3)	28	21	-0.03	-0.01	-0.23	-0.54
					Inferior	11 (±1.8)	24 (±2.8)	33	23	-0.07	-0.13	-0.32	-0.39

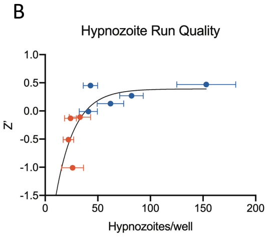

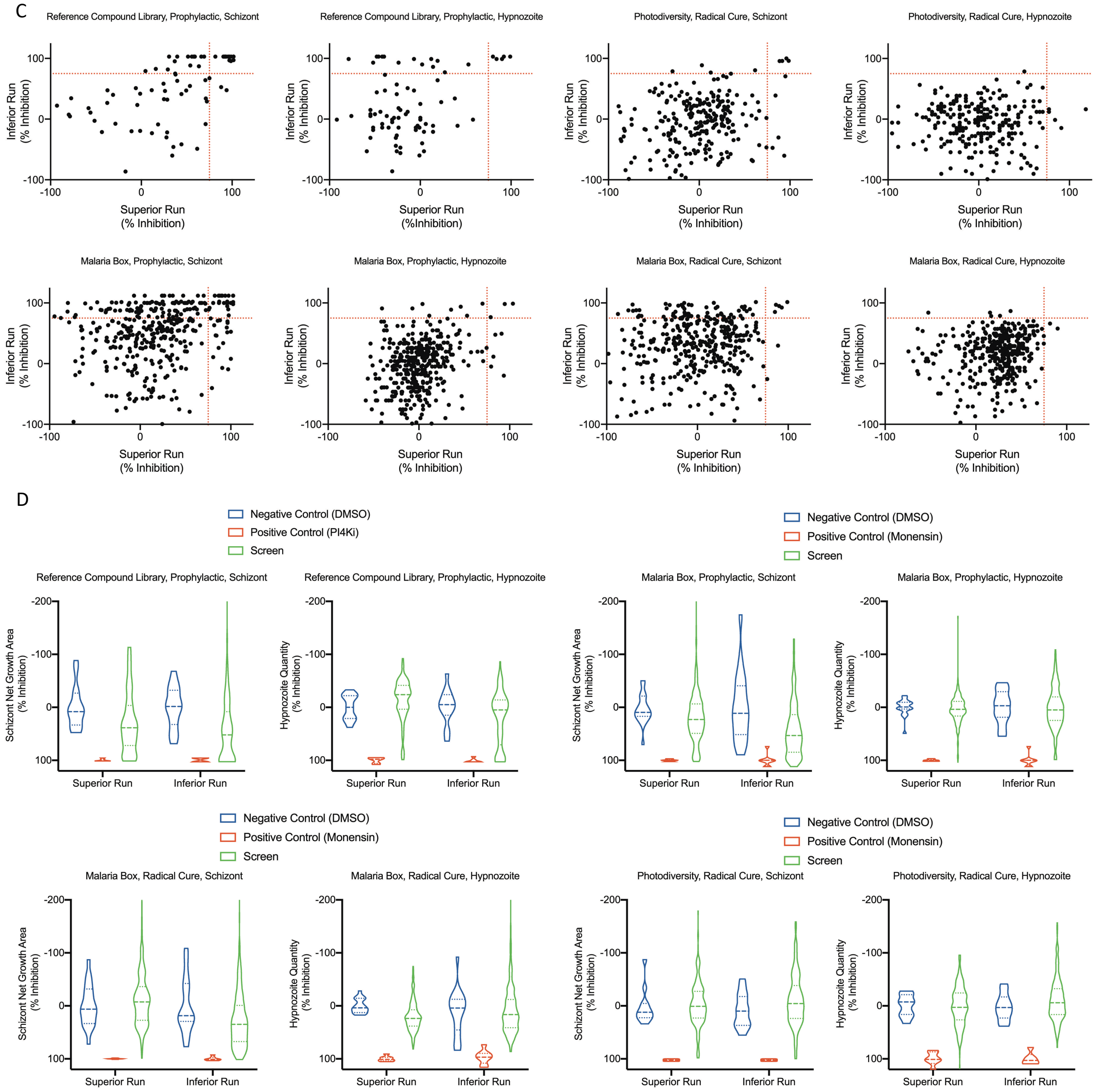

Figure S3. Multi-run analyses and putative hit consistency. A) Infection rate and statistical metrics from every Single Point run. Sch, Schizont; Hyp, Hypnozoite; SD, standard deviation; CV, coefficient of variance; Z', Z' factor between positive and negative controls; Z, Z score between the inhibition of screen wells and the positive control. B) Nonlinear regression of Z' factor as a function of infection rate. A minimum of about 40 hypnozoites per well is needed to obtain a positive Z' factor. Bars represent SD. C) The Reference Compound Library, Photodiversity Library,

and Malaria Box were ran twice; the higher-quality run (as measured by Z' factor as well as absence of confounding results such as edge effects or plate gradients) was designated as the "Superior Run" while the other was designated as the "Inferior Run." Red dotted lines indicate 75% inhibition for reference. Paired data for each compound from both runs were plotted together to show which compounds were consistently putative hits (top right area), versus compounds which were likely false positive due to poor run quality (top left area and bottom right area). D) Violin plots from the negative control wells (blue) positive control wells (red), and screen wells (green) from each set of Superior and Inferior runs. The positive controls perform the same regardless of run quality, resulting in a consistent average at and narrow deviation around 100% inhibition. Conversely, the negative control and screen well average and deviation, as well as the separate between positive and negative control means, are dependent on run quality. Solid line, average; dotted lines, 25% and 75% quartiles.

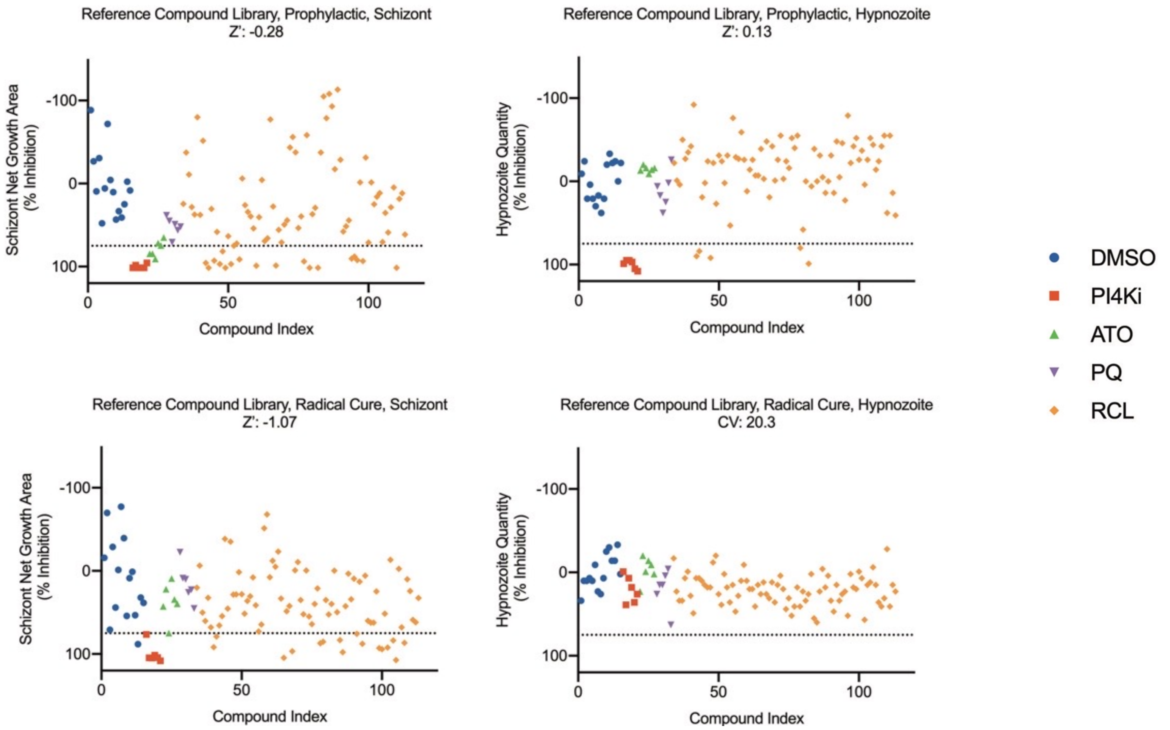

Figure S4. Single point activity from the Reference Compound Library. The net schizont growth area per well for both the Prophylactic and Radical Cure runs, as well as the hypnozoite quantity per well from the Prophylactic run, were normalized to the PI4Ki controls; Z' factor is shown for each run. Hypnozoite quantity per well from the Radical Cure run was normalized to DMSO controls and percent inhibition was calculated as 100%-(% remaining hypnozoites); the CV (%) for hypnozoite quantity in DMSO wells is shown. Dotted line represents 75% inhibition for reference. Legend: DMSO, negative control; PI4Ki, MMV390048 positive control; ATO, atovaquone reference compound; PQ, primaquine reference compound; RCL, Reference Compound Library.

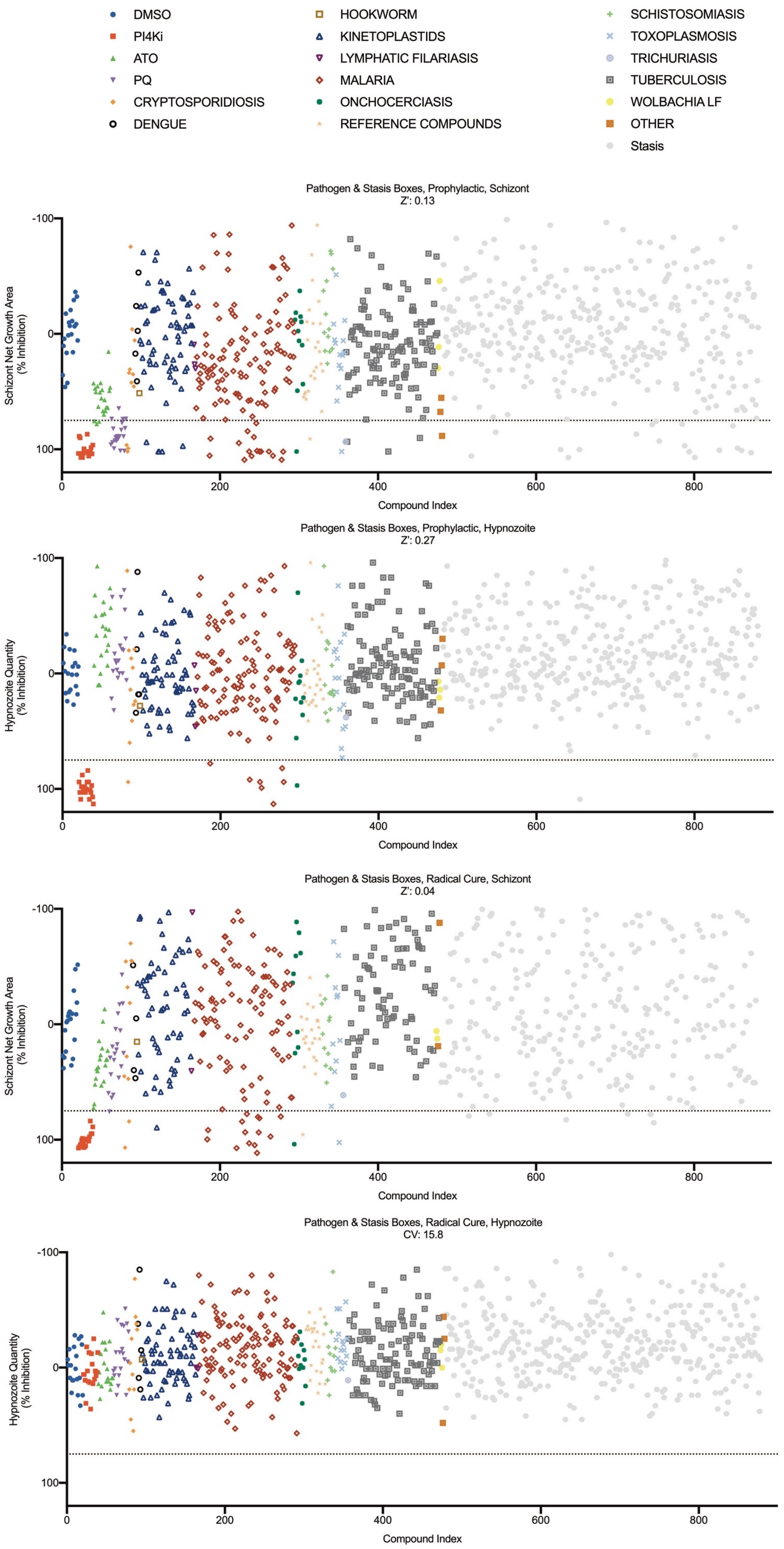
DMSO
PI4Ki
ATO
PQ
CRYPTOSPORIDIOSIS
DENGUE
HOOKWORM
KINETOPLASTIDS
LYMPHATIC FILARIASIS
MALARIA
ONCHOCERCIASIS
REFERENCE COMPOUNDS
SCHISTOSOMIASIS
TOXOPLASMOSIS
TRICHURIASIS
TUBERCULOSIS
WOLBACHIA LF
OTHER
Stasis
Pathogen & Stasis Boxes, Prophylactic, Schizont
Z': 0.13
Schizont Net Growth Area (% Inhibition)
-100
0
100
0
200
400
600
800
Compound Index
Pathogen & Stasis Boxes, Prophylactic, Hypnozoite
Z': 0.27
Hypnozoite Quantity (% Inhibition)
Compound Index
Pathogen & Stasis Boxes, Radical Cure, Schizont
Z': 0.04
Schizont Net Growth Area (% Inhibition)
Compound Index
Pathogen & Stasis Boxes, Radical Cure, Hypnozoite
CV: 15.8
Hypnozoite Quantity (% Inhibition)
Compound Index

Figure S5. Single point activity from the Pathogen and Stasis Boxes. The Pathogen Box and Stasis Box were merged and ran together. The net schizont growth area per well for both the Prophylactic and Radical Cure runs, as well as the hypnozoite quantity per well from the Prophylactic run, were normalized to the PI4Ki controls; Z' factor is shown for each run. Hypnozoite quantity per well from the Radical Cure run was normalized to DMSO controls and percent inhibition was calculated as 100%-(% remaining hypnozoites); the CV (%) for hypnozoite quantity in DMSO wells is shown. Dotted line represents 75% inhibition for reference. Legend: DMSO negative control; PI4Ki, MMV390048 positive control; ATO, atovaquone reference compound; PQ, primaquine reference compound; PB compounds were characterized by disease set and are represented with different points as indicated; Stasis, SB compounds.

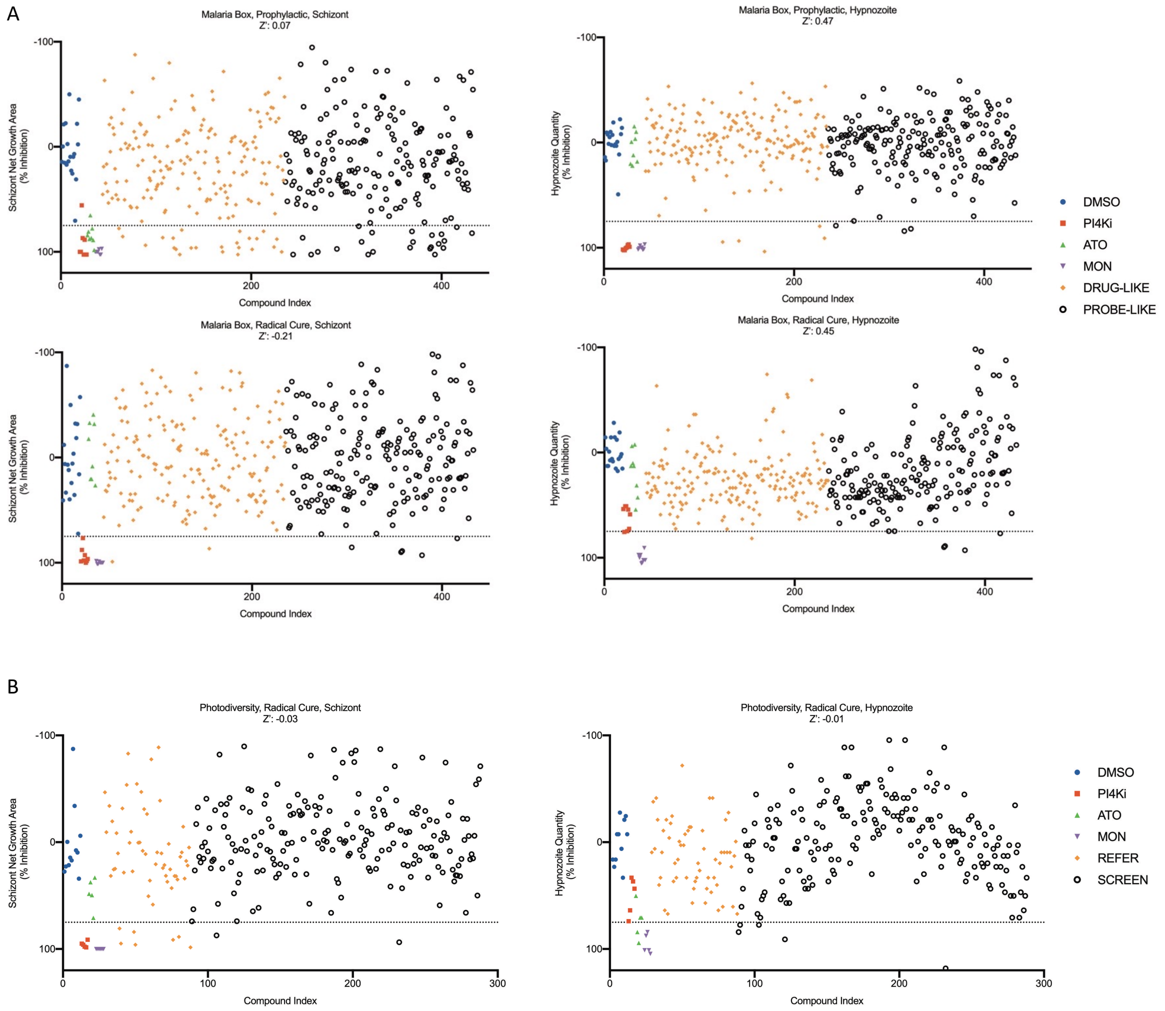

Figure S6. Single point activity from the Malaria Box and Photodiversity Library. The net schizont growth area per well for both the Prophylactic and Radical Cure runs, as well as the hypnozoite quantity per well from for both the Prophylactic and Radical Cure runs, were normalized to the monensin controls; Z' factor is shown for each run. Dotted line represents 75% inhibition for reference. Legend: DMSO negative control; PI4Ki, MMV390048 reference compound; ATO, atovaquone reference compound; MON, monensin positive control; Malaria Box compounds were characterized as either Probe-like or Drug-like and are represented with different points as indicated; REFER, the Photodiversity Library contained 60 reference compounds which are represented with different points as indicated.

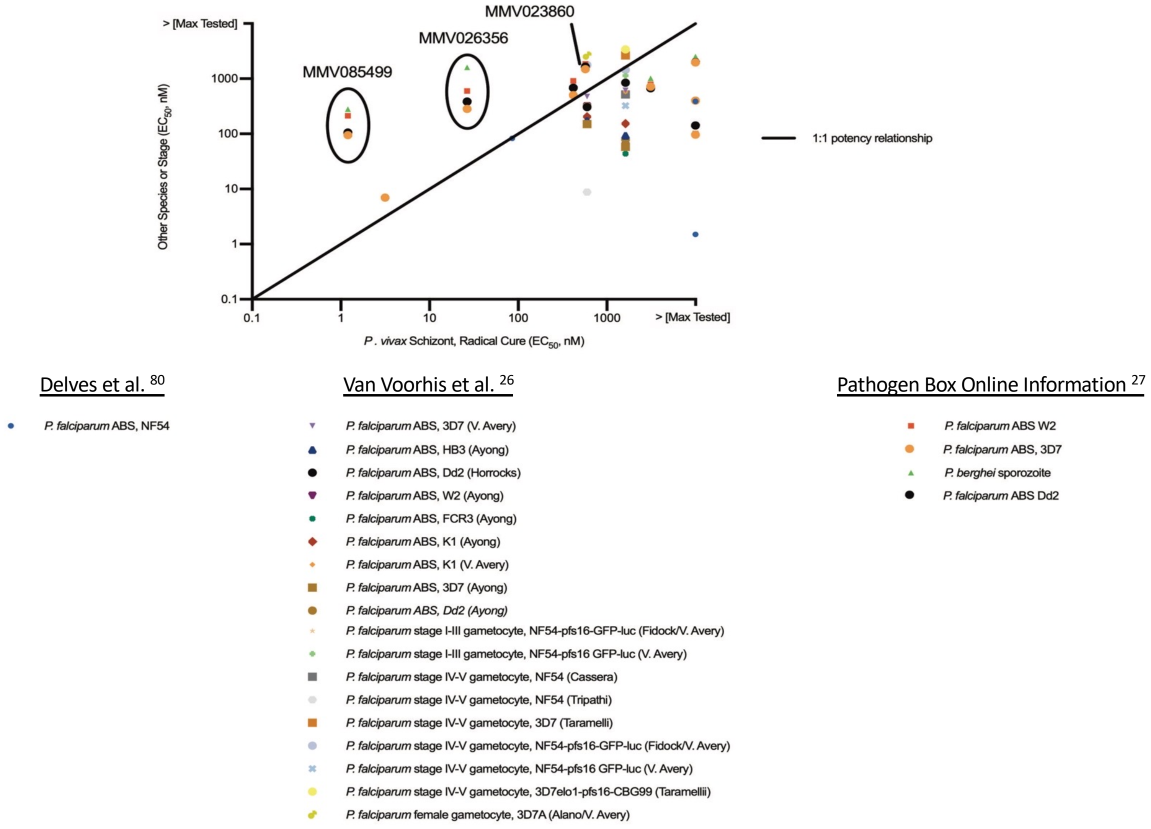

Figure S7. Cross-comparison of potency against *P. vivax* late schizonts and other *Plasmodium* species and lifecycle stages. Data were derived from previously-published data or information available with the Pathogen Box. Markers above black line indicate more potency against *P. vivax* late schizonts, markers below black line indicate less potency against *P. vivax* late schizonts. ABS: asexual blood stage.

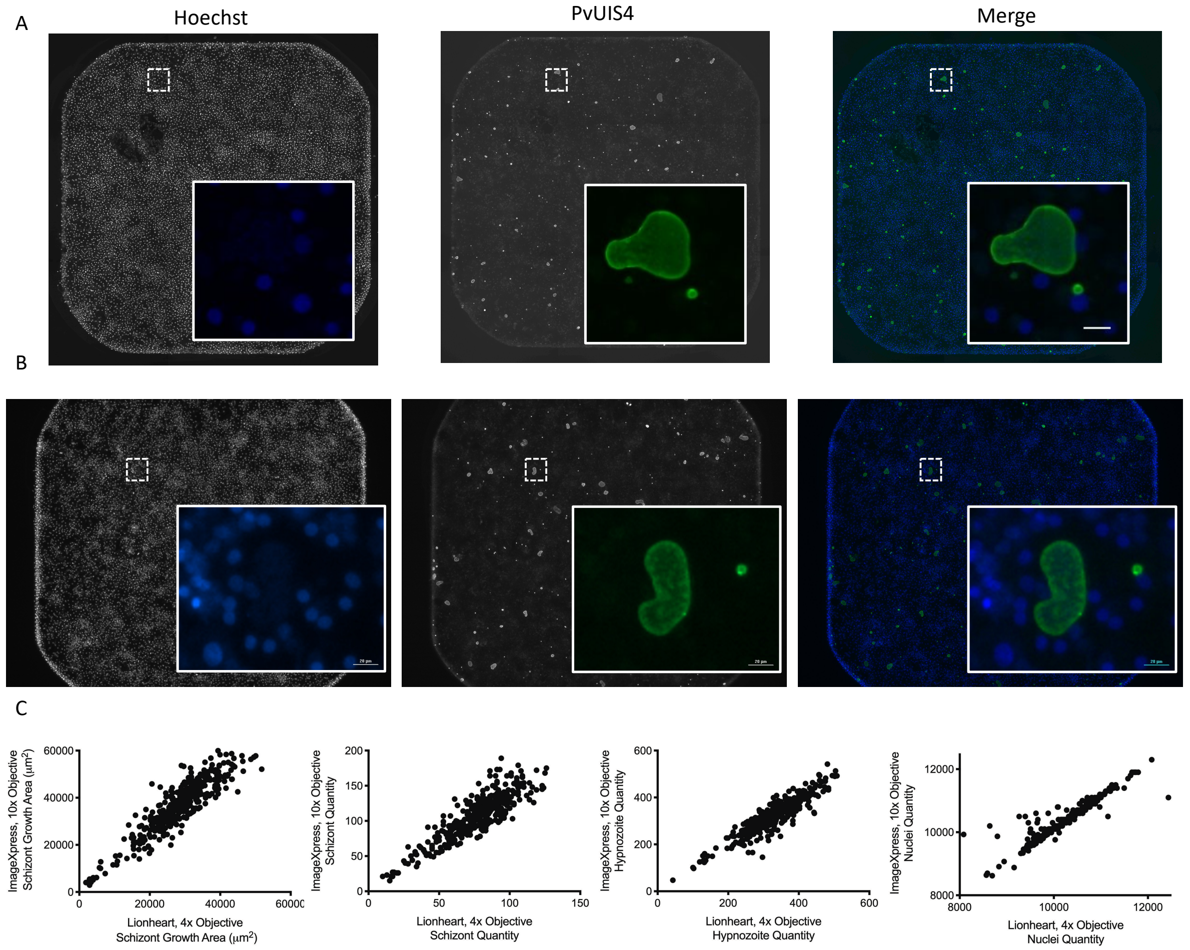

Figure S8. High Content Imaging methodology and validation. High Content Imaging (HCI) was first performed on an ImageXpress (IMX) Micro Confocal with a 10x objective and then with a Lionheart with a 4x objective. A) Representative image from a single well of a 384-well plate, stitched from 9 fields of view imaged with a 10x objective on an IMX. Inset: digitally zoomed image from area marked by dotted white line. B) Representative image from a single well of a 384-well plate, stitched from 4 fields of view imaged with a 4x objective on a Lionheart. Inset: digitally zoomed image from area marked by dotted white line. Bar represents 20 µm. C) HCI data obtained from an IMX and Lionheart was compared for several assay plates, including one with a very high infection rate of over 500 parasites per well (shown). Each point represents the PvLS parasite growth metrics or nuclei count for a single well.
